# Population Pharmacokinetics, Efficacy Exposure-response Analysis, and Model-based Meta-analysis of Fenebrutinib in Subjects with Rheumatoid Arthritis

**DOI:** 10.1007/s11095-019-2752-y

**Published:** 2020-01-06

**Authors:** Phyllis Chan, Jiajie Yu, Leslie Chinn, Marita Prohn, Jan Huisman, Brett Matzuka, William Hanley, Katie Tuckwell, Angelica Quartino

**Affiliations:** 1 Clinical Pharmacology, Genentech Inc., South San Francisco, California USA; 2 qPharmetra, Nijmegen, Netherlands; 3 qPharmetra, Cary, North Carolina USA; 4 Former Genentech employee, currently of Seattle Genetics, South San Francisco, California USA; 5 Clinical Sciences, Early Clinical Development, Genentech, South San Francisco, California USA

**Keywords:** Bruton’s tyrosine kinase (BTK) inhibitor, exposure-response, model-based meta-analysis, population pharmacokinetics, rheumatoid arthritis

## Abstract

**Purpose:**

Fenebrutinib (GDC-0853), a Bruton’s tyrosine kinase (BTK) inhibitor was investigated in a Phase 2 clinical trial in patients with rheumatoid arthritis (RA). Our aim was to apply a model-informed drug development (MIDD) approach to examine the totality of available clinical efficacy data.

**Methods:**

Population pharmacokinetics (popPK) modeling, exposure-response (E-R) analysis, and model-based meta-analysis (MBMA) of fenebrutinib were performed based on the Phase 2 data.

**Results:**

PopPK of fenebrutinib after oral administration was described using a 3-compartment model with linear elimination and a flexible absorption transit compartment model. Healthy subjects had a 52% higher apparent clearance than patients. E-R analyses based on longitudinal ACR20, ACR50, and ACR70 and DAS28 (CRP) data modeled fenebrutinib effect with an E_max_ function, and an efficacy plateau was achieved within the exposure range obtained in the Phase 2 clinical trial. Based on literature data, a summary-level clinical efficacy database was constructed, and MBMA determined ACR20, ACR50, and ACR70 responder rates in the placebo and adalimumab arms of the Phase 2 clinical trial were found to be consistent with historical data for these treatments.

**Conclusions:**

Our multi-pronged approach applied MIDD to maximize knowledge extraction of efficacy data and enabled robust interpretation from a Phase 2 clinical trial.

**Electronic supplementary material:**

The online version of this article (10.1007/s11095-019-2752-y) contains supplementary material, which is available to authorized users.

## Introduction

### Model Informed Drug Development (MIDD)

In 2017, the sixth iteration of the Prescription Drug User Fee Act (PDUFA VI) was authorized as part of the FDA Reauthorization Act (FDARA). Among its goals was to augment the Agency’s expertise in model-informed drug development (MIDD) approaches and support the evaluation of model-based strategies used to guide development efforts (1).

MIDD approaches can be extremely informative to drug development: they can be used to characterize the exposure-response (E-R) relationship of new drugs, especially when the relationship is complex and its interpretation challenging, and to support selection of the appropriate dose and/or regimen for an upcoming clinical trial. These approaches result in more powerful inferences than other, non-integrated analyses as they help to fully identify various aspects of the E-R relationship and maximize knowledge when harvesting from the drug’s proof of concept study by examining the totality of clinical efficacy and safety data. Drug development in rheumatoid arthritis (RA) patients can be used to illustrate the use of MIDD approaches (2) in particular, where a large number of studies showing the efficacy of therapy across various mechanisms of action have been published, thus providing the ability to pool data and make salient comparisons.

### Bruton’s Tyrosine Kinase (BTK) and Fenebrutinib

RA is an autoimmune disorder, and although several therapeutics are approved for the treatment of patients with an inadequate response to methotrexate (MTX) or tumor necrosis factor inhibitors (anti-TNF) (3–9), some patients become refractory to currently available therapeutics and may benefit from therapy utilizing a different mechanism of action (6,8–12).

Bruton’s tyrosine kinase (BTK) is a nonreceptor Tec family tyrosine kinase broadly expressed in hematopoietic cells (except T cells) and plays a crucial role in signaling through the B cell antigen receptor and the Fcγ receptor (FcγR) in B cells and myeloid cells, respectively (13–16). Therefore, the inhibition of BTK represents an attractive potential therapeutic approach for the treatment of immunological disorders such as RA or systemic lupus erythematosus (SLE) (15–20), in which B cells and myeloid cells induce or sustain an excessive autoimmune response. However, currently there is no approved BTK-targeted therapy for such chronic autoimmune indications.

Fenebrutinib (GDC-0853, RG7845) is an orally administered BTK inhibitor that is highly selective and noncovalent, leading to reversible binding, intended to block B cell proliferation and the resulting excessive immune response seen in autoimmune disorders (21). Fenebrutinib has previously been evaluated in healthy subjects (22) and patients with resistant B cell lymphoma or chronic lymphocytic leukemia (23).

The pharmacokinetics (PK) of fenebrutinib was characterized previously in healthy subjects in a Phase 1 trial, and plasma concentrations peaked 1–3 h after oral administration and declined thereafter, with a steady-state half-life ranging from 4.2–9.9 h (22). In the Phase 1 trial, fenebrutinib plasma exposures were found to increase approximately dose-proportionally with modest accumulation following twice daily dosing. Additionally, dose- and concentration-dependent inhibition of BTK was observed, and pharmacokinetic/pharmacodynamic (PK/PD) models were developed using the PD biomarkers data to describe the treatment effect of fenebrutinib on BTK inhibition, as assessed by BTK autophosphorylation on circulating B cells and basophils in healthy volunteers. Subsequent simulations conducted with these PK/PD models suggest that a once-daily dosing regimen would maintain steady-state plasma concentrations associated with a high degree of CD63 inhibition over the entire dosing interval.

Fenebrutinib is currently being investigated in patients with RA, chronic spontaneous urticaria, and SLE. To fully understand the treatment effect of fenebrutinib in patients with RA, an MIDD approach was implemented by investigating the totality of clinical efficacy data from a Phase 2 trial through the integration of population pharmacokinetics (popPK) modeling, E-R analysis, and model-based meta-analysis (MBMA) of fenebrutinib.

## Materials and Methods

### Trial Design

GA29350 (ANDES, ClinicalTrials.gov Identifier: NCT02833350) was a multicenter, Phase 2, randomized, double-blind, placebo-controlled, parallel-group, dose-ranging trial to evaluate the efficacy and safety of fenebrutinib in patients with moderate to severe active RA and an inadequate response to previous MTX therapy (cohort 1) or anti-TNF therapy (cohort 2) (24,25). All patients were seropositive for either rheumatoid factor (RF) or anti-citrullinated peptide antibody, or had a history of seropositivity upon entry into the trial and were on stable doses of MTX during the trial.

In cohort 1, 480 patients were enrolled, and were randomly assigned to 1 of 5 parallel treatment arms (3 fenebrutinib arms, 1 placebo arm, and 1 adalimumab arm). In the fenebrutinib and placebo arms, subjects received 50 mg QD, 150 mg QD, or 200 mg BID fenebrutinib tablets or placebo tablets, together with subcutaneous (SC) placebo injections every other week for 12 weeks (*n* = 40 in the 50 mg arm, and *n* = 109 to 111 in each of the other 4 arms). In the active comparator arm, patients received adalimumab 40 mg SC every other week and placebo tablets for 12 weeks. In cohort 2 (*n* = 98 enrolled), patients received 200 mg fenebrutinib BID or placebo tablets for 12 weeks.

Fenebrutinib dose selection for the Phase 2 trial was based on its safety profile, PK properties, and the expectation that plasma concentrations achieved would reach 70 to 90% BTK inhibition over the dosing interval in the majority of patients. Three dose levels were selected for cohort 1 to obtain a broad range of fenebrutinib exposures, with minimal exposure overlap among the dose groups, to allow a thorough investigation of E-R in patients. As the lowest fenebrutinib dose (50 mg QD) in the trial was not expected to achieve maximal efficacious response, a reduced number of subjects were assigned to this dose group.

Fenebrutinib plasma concentrations were determined by high performance liquid chromatography with tandem mass spectrometry (MS/MS) detection (Covance Laboratories Inc., USA). Calibration plots were linear throughout the range of 0.5 to 500 ng/mL (lower and upper limits of quantification, respectively) for fenebrutinib in human plasma. Relative standard deviation of precision was <7.8%, and the accuracy of the method was between 91.3% and 102.7%.

### Population Pharmacokinetic (popPK) Analysis

Fenebrutinib plasma concentrations from subjects providing all available PK data after tablet administration in the clinical studies were integrated into the popPK analysis. The model was characterized initially in healthy subjects (22) from the Phase 1 multiple ascending dose (MAD) (GA29347) and a Phase 1 relative bioavailability/food effect/drug-drug interaction (GP29832, ClinicalTrials.gov Identifier: NCT02699710) studies, and later updated to incorporate the Phase 2 data from the three fenebrutinib-treated arms in patients with RA. PK sampling schedules of the 3 studies included in the popPK analysis are presented in Table S1.

The popPK model was developed by introducing features in the order of increasing complexity, beginning with very simple models (e.g. one-compartment with first-order elimination), and continuing until further improvement in model fit was not supported by the data.

Covariate analysis first investigated the effects of pre-defined demographic, laboratory, prognostic, and treatment covariates on all the model parameters by using univariate screening. If >10% of a covariate was missing, the covariate was not included in the popPK analysis. Otherwise, the value of missing covariate observations was imputed as the median of the remaining values from an appropriate sub-population. For example, the missing baseline body weight value was imputed as the gender-adjusted median body weight of the remaining subjects. For categorical covariates, the most frequently occurring value was imputed for subjects with missing values in each trial. Concomitant proton pump inhibitor (PPI) and food were categorized as time-varying covariates, whereas the other categorical covariates were considered as baseline (non-time-varying) covariates. Next, covariates selected from the univariate screening (*p* value <0.05) were included in the evaluation of a full model followed by backwards elimination (*p* value <0.01). Effects of continuous covariates were incorporated into the model using the power function and normalized using the median of the covariate. Effects of categorical covariates were parameterized as proportional effects in the model, in which the fractional change on a model parameter was estimated, and the typical category constituted the largest proportion in the analysis population.

The final model was determined on the basis of objective function value, physiological plausibility of model parameter estimates, numerical convergence success, parameter estimate precision (relative standard errors <50%), and visual predictive check (VPC) plots. Goodness-of-fit plots were produced to verify the general agreement of fitted values with observed data. Prediction-corrected visual predictive check (pcVPC) was performed by normalizing the observed and simulated dependent variable based on the typical population prediction for the median independent variable in the bin (26). The pcVPC was stratified to evaluate the covariate effects of interest, whenever applicable.

In addition to exploring the effects of covariates on the intersubject variability of PK of fenebrutinib, the popPK model was also used to generate empirical Bayes estimates (EBE; individual posthoc) for exposure, in terms of maximum concentration (C_max_), trough concentration (C_min_), and total daily area-under-the-concentration-time-curve (AUC) at steady state as predicted using nominal dose due to the high patient compliance in the trial (dose intensity rate ≥ 99.3%/arm/cohort), for the E-R analysis.

Model development was carried out using first order conditional estimation with interaction (FOCE-I) as implemented in NONMEM (version 7.3, ICON Development Solutions, Ellicott City, MD). Post-processing of NONMEM analysis results was carried out in R (version 3.2.2, R Development Core Team, 2008).

### Exposure-Response (E-R) Analysis

Treatment responses in RA clinical trials are commonly evaluated by the American College of Rheumatology improvement criteria for 20, 50, and 70% threshold values (ACR20, ACR50, and ACR70, respectively), which is a binary response variable, and by the Disease Activity Score with 28-joint counts (DAS28), which is a continuous variable (27,28). Both types of endpoints can be measured repeatedly during the course of the treatment period. The proportion of patients achieving ACR50 responses and analysis of longitudinal DAS28 (CRP) (DAS based on 28 joints and C-reactive protein value) were evaluated as the primary, key secondary, and secondary endpoints in the Phase 2 trial, and therefore, were of primary interest among clinical efficacy endpoints modeled for fenebrutinib. The E-R analyses aimed to establish the relationship between patient-specific fenebrutinib exposure with clinical efficacy endpoints of interest.

### ACR20, ACR50, and ACR70

Modeling of ACR20, ACR50, and ACR70 responses in individual patients following treatment with fenebrutinib used observations from cohorts 1 and 2 from the Phase 2 trial. The E-R analysis dataset consisted of a total of 7005 observations (2335 each for ACR20, ACR50, and ACR70) from 467 patients. Observations were on days 7, 14, 28, 56, and 84 after the initiation of study treatment. Study dropout rate was low, with a majority of patients completing the 12-week trial with ≥90% completion rate/arm/cohort. Non-responder imputation was used for patients who discontinued prior to week 12, and for whom an ACR response could not be determined. Given the number of patients and use of imputation, there were 467 observations available on each observation day. Study treatments included placebo and fenebrutinib doses of 50 mg QD, 150 mg QD, and 200 mg BID.

Fenebrutinib exposure metrics of C_max_, C_min_, and AUC at steady state as the driver for the E-R relationship were explored. Graphics created with R were used to view the time course of fractions of ACR20, ACR50, and ACR70 responders, placebo effect, and treatment effect based on fenebrutinib exposure.

An E-R analysis was conducted with NONMEM, using Laplacian conditional estimation with −2 times the log of the likelihood (−2LL). Simultaneous modeling (29) of ACR20, ACR50, and ACR70 and all time points reflected the analysis decision to utilize the benefits of pooling data across highly interrelated observations. The E-R model considered the longitudinal measurements as a function of time, placebo effect, and fenebrutinib exposure:

$$ {\displaystyle \begin{array}{c} logit\ of\  ACR= Baseline+ Time\ effect+ Covariate\ effect+ Markovian\ element\\ {} Time\ effect=\frac{Tmax\times tim{e}^{Hill}}{tim{e}^{Hill}+{T_{50}}^{Hill}}\\ {} Covariate\ effect= Geographic\ region+ Rheumatoid\ factor+\frac{Fenebrutinib\  AUC}{Fenebrutinib\  AUC+E{C}_{50}}\end{array}} $$

where Tmax is the maximum placebo effect over time, T50 is the time associated with 50% of placebo effect, and EC50 is the fenebrutinib exposure (in terms of AUC) associated with 50% of drug effect. Finally, an analysis of covariate effects on the endpoints was performed, and simulation (*n* = 1000) was conducted based on the final model and summarized using posterior predictive check (30).

### DAS28 (CRP)

Modeling of the E-R relationship between fenebrutinib exposure and DAS28 (CRP) response included observed data from cohorts 1 and 2 of the Phase 2 trial. The analysis dataset consisted of 2676 observations from 467 patients measured on days 0, 7, 14, 28, 56, and 84 after the initiation of study treatment. There were 467 observations at baseline, decreasing to 424 observations on day 84. The data utilized last observation carried forward (LOCF) imputation but excluded withdrawal. As was done for E-R analysis in ACR20, ACR50, and ACR70, patient-specific predictions of exposure metrics in terms of steady state C_max_, C_min_, and AUC from the popPK model were used to enable E-R modeling. In addition, the data were explored graphically prior to modeling, and an E-R analysis was performed using longitudinal DAS28 (CRP) data. Model development was conducted using first-order conditional estimation with interaction (FOCE-I) as implemented in NONMEM. The final model fit the observed data as a function of time, placebo effect, and fenebrutinib exposure. No covariate analysis was performed due to the supportive and exploratory nature of using a second efficacy endpoint, and simulation (*n* = 1000) was conducted based on the final model and summarized using visual predictive check.

### Model-Based Meta-Analysis (MBMA) Modeling of ACR20, ACR50, and ACR70

An efficacy and safety meta-analysis database was constructed in July 2017 using publicly available data of randomized trials in RA, based on the guidelines in the Cochrane Handbook for Systematic reviews. For this, a systematic review of publicly available data from the PubMed, Cochrane Library, and Embase databases was conducted using the search term “rheumatoid arthritis”. Furthermore, an existing database that contained data published in or before 2012 (30) was augmented using an additional, systematic, and quality-controlled procedure to search for relevant published studies, extract their data, and add to the existing database yielding pertinent data published through 2017.

The summary data of the database were explored systematically to determine the amount of data available for each treatment, efficacy and safety endpoint across trials, distribution of mean patient characteristics, and to graphically view the time course of longitudinal endpoints, placebo effect, active treatment effect from each of the comparator treatments, and the effects of patient and trial characteristics. Response rates of ACR20, ACR50, and ACR70 in terms of the proportion of patients achieving specified thresholds were the most prevalent efficacy endpoints in the database, and therefore, were chosen as the variables for model development using MBMA. Because of the relatively few adverse events observed in the fenebrutinib Phase 2 trial in RA (24), MBMA for safety events, such as serious infections, was not conducted.

The model simultaneously fit ACR20, ACR50, and ACR70 longitudinal data, and a non-linear mixed effects (NLME) estimation method was carried out using R. This hierarchical regression model used maximum likelihood estimation, and multiple levels of heterogeneity were described as mixed effect variability terms accommodating between-trial and within-trial variability. A residual variability term was used to account for the unexplained deviation from fixed effects (31). The effects of time, placebo treatment, active drug dose, and additional covariates were considered during model development. For treatments where dose-ranging data were available, the potential dose-response relationships were tested.

The adequacy of model fits across trials was evaluated by visually inspecting time course plots of observed distributions *vs.* predicted values. Model simulations were performed using fixed and random effect estimates from the final MBMA model to predict the treatment effects over time at hypothetical doses of fenebrutinib comparing to the approved drug dose levels, as well as to further evaluate difference in treatment response rates among various patient populations in RA.

## Results

### Population Pharmacokinetic (popPK)Modeling

The popPK analysis dataset consisted of 130 males and 255 females, between 18 and 75 years of age, and baseline body weights ranging from 38 to 153 kg. The demographics of the subjects included in the popPK analysis are summarized in Table S2. No imputation of PK values was performed, and records with missing PK or time values were omitted from the analysis. In total, 506 (11%) out of 4565 observations were reported as having concentration below the lower limit of quantification (BLQ), and were excluded from the analysis without further assessment of their impact on parameter estimates. Predefined set of clinical and demographic covariate factors (Table S2) were investigated during popPK model development to assess their influence on the PK characteristics. The analysis dataset did not include C-reactive protein (CRP) values from healthy subjects in the Phase 1 multiple ascending dose (MAD) trial or race information from the Phase 1 relative bioavailability/food effect/drug-drug interaction trial.

The final popPK model was a 3-compartment model with linear elimination from the central compartment. Its parameter estimates are shown in Table I. A flexible transit compartment absorption model (TCAM) was used to describe the fenebrutinib absorption (32). The final model included effects of PPI concomitant administration, food intake, and formulation on mean transit time (MTT) between absorption compartments, number of transit compartments (NTR), and relative extent of absorption (F1). In addition, concomitant administration of PPI, age, and subject status (i.e. healthy subject or RA patient) were found to affect apparent clearance (CL/F). The residual variability was described using a proportional error model that was assumed to decrease with time via an exponential model for the Phase 1 studies and constant for Phase 2 trial, due to the sparse sampling in the Phase 2 clinical trial.

**Table I:** Parameter Estimates of the Final popPK Model

Parameter	Alias	Estimate	Relative SE (%)	95% CI	Shrinkage (%)
θ_1_	CL/F: Apparent systemic clearance (L∙h^−1^)	19.5	.....	(18.2–20.9)	
θ_2_	V1/F: Apparent central volume of distribution (L)	381	.....	(333–437)	
θ_3_	V2/F: Apparent peripheral volume of distribution (L)	284	.....	(254–318)	
θ_4_	Q1/F: Apparent Intercompartmental clearance (L∙h^−1^)	52.8	.....	(44.1–63.2)	
θ_5_	V3/F: Apparent second Peripheral volume of distribution (L)	273	.....	(222–336)	
θ_6_	Q2/F: Apparent second intercompartmental clearance (L∙h^−1^)	4.47	.....	(3.73–5.36)	
θ_7_	NTR: Number of transit compartments	14.9	.....	(13.0–17.1)	
θ_8_	MTT: Mean transit time (h)	0.849	.....	(0.755–0.954)	
θ_9_	Proportional residual error (%)	1.94	.....	(1.29–2.92)	
θ_11_	Food effect on MTT	1.43	.....	(1.22–1.68)	
θ_12_	PPI effect on MTT	0.835	.....	(0.692–1.01)	
θ_13_	PPI and food effect on MTT	2.26	.....	(1.78–2.86)	
θ_14_	PPI effect on F1	0.657	.....	(0.568–0.759)	
θ_15_	PPI and food effect on F1	0.693	.....	(0.611–0.785)	
θ_16_	Food effect on NTR	0.864	.....	(0.45–1.66)	
θ_17_	Tablet effect on NTR	0.049	.....	(0.0287–0.0838)	
θ_18_	Residual error rate in healthy volunteers	1.94	.....	(1.53–2.46)	
θ_19_	Maximum residual error in healthy volunteers	6.66	.....	(4.21–10.6)	
θ_20_	PPI effect on CL/F	0.663	.....	(0.650–0.675)	
θ_21_	Proportional residual error in patients	0.390	.....	(0.372–0.408)	
θ_22_	Age effect on CL/F	−0.161	41.4	(−0.291 - -0.0304)	
θ_23_	Healthy volunteer effect on CL/F	1.52	.....	(1.27–1.82)	
ω_1.1_	ω^2^_CL/F_	0.0732	14.9	(0.0518–0.0946)	27.3
ω_2.2_	ω^2^_V1/F_	0.100	22.6	(0.0558–0.145)	54.4
ω_8.8_	ω^2^_MTT_	0.0861	29.7	(0.0360–0.136)	58.1
ω_9.9_	ω^2^_F1_	0.131	17.1	(0.0872–0.175)	31.9
ω_10.1_	ω^2^_IOV on F1_	0.299	5.30	(0.268–0.330)	26.0

Log-transformed parameters have been back-transformed and thus relative SE (%) is not represented, instead 95% CI is appropriate

*popPK* population pharmacokinetics, *SE* standard error, *CI* confidence interval, *PPI* proton pump inhibitor, *F1* absorption

The final popPK model (Model S1) captured the observed data well and adequately described the overall fenebrutinib concentration *versus* time profiles. The performance and adequacy of the model were demonstrated using GOF plots (Fig. S1) and pcVPC plots (Fig. S2). Median individual post hoc exposures among the three fenebrutinib dose groups investigated in the Phase 2 trial were well separated (Fig. S3). The epsilon-shrinkage value was 12.5%.

Statistically significant covariates included the impact of subject status, age, and concomitant administration of PPI on apparent clearance. Healthy subjects in the two Phase 1 studies had a 52% higher apparent clearance than patients with RA in the Phase 2 trial. Additionally, apparent clearance (CL/F) decreases with age (15.2% lower in a 75-year-old patient compared to a 25-year-old patient), and concomitant administration of PPI (decreases by 33.7%).

### Exposure-Response (E-R) Modeling

#### ACR20, ACR50, and ACR70

The efficacy of fenebrutinib as defined by the probability of achieving ACR20, ACR50, and ACR70 was described by a logistic model that represented effects of treatments as a function of time, fenebrutinib exposure at steady state, and patient characteristics. The time component was modeled as a sigmoidal E_max_ function to represent the increase in efficacy to a maximum over time. The fenebrutinib effect on response was modeled as an E_max_ function driven by fenebrutinib exposure as measured by C_max_, C_min_, or AUC, and the statistical significance of incorporating each exposure metric into the E-R model was similar in terms of *p*-values. Steady state AUC was chosen as the driver for efficacy, based on exploratory results, and prior E-R modeling knowledge in RA (33,34). Separate E_max_ functions to describe the E-R relationship for each of ACR20, ACR50, and ACR70 probability were tested, but it was seen that a single E_max_ term for all three ACR thresholds adequately captured the data. Additionally, a Markovian element was added to reflect the inherent serial correlation in the data (29). It is notable that by definition, at time zero, all patients are non-responders, and thus observed baseline measurement is zero. However, during model development, it was found that including estimated baselines in the model improved the fit at later time points substantially, without unduly adversely affecting the model predictions at time zero, where it is understood that the actual result is zero probability. Therefore, separate baseline terms were estimated for each of ACR20, ACR50, and ACR70 probability achievement, and an inter-individual variability term was added on baseline (Model S2).

A clear E-R relationship was observed between fenebrutinib exposure in terms of steady state AUC and the probability of achieving ACR20, ACR50, and ACR70. A dropout model was not incorporated due to the high completion rate (≥90%) of the Phase 2 trial. Table S3 shows the summary statistics of the list of predefined covariates explored for the E-R analysis. The subsequent univariate screening, followed by full model formation and then backward elimination, found that baseline rheumatoid factor (RF) level was a statistically significant covariate. Parameter estimates for the E-R model using ACR20, ACR50, and ACR70 data are shown in Table S4.

**Fig. 1:**
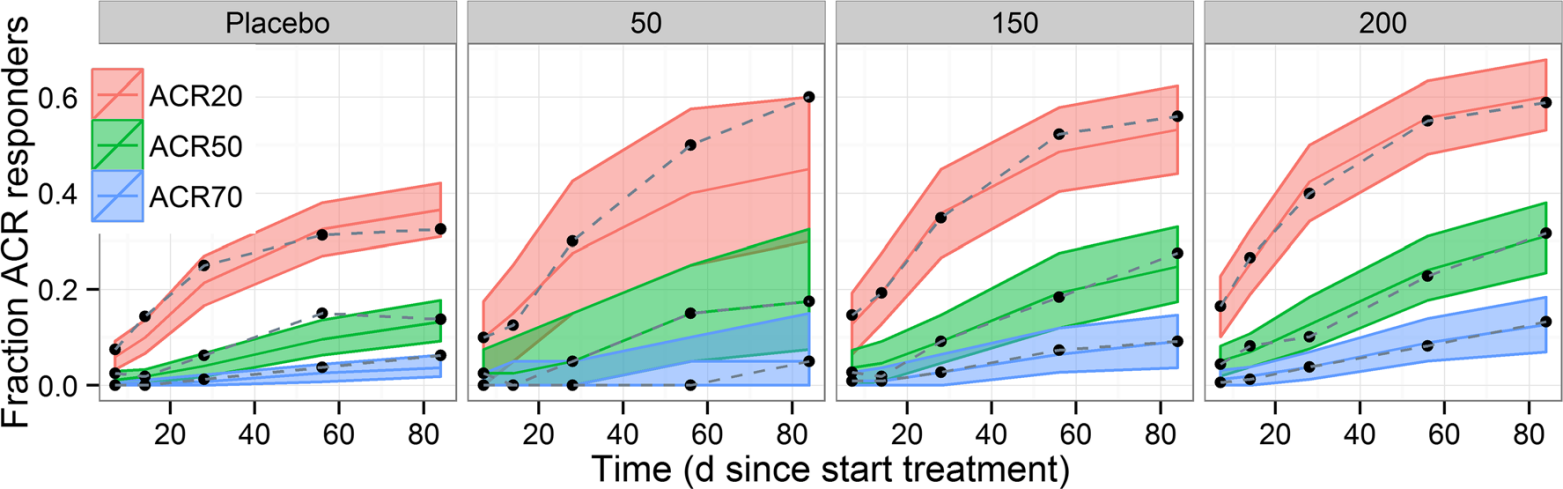
Posterior predictive check for exposure-response model of ACR20, ACR50, and ACR70 – time course by fenebrutinib dose. Black dots: observed responder fraction; bands: 90% prediction interval; red, green, blue middle lines: median prediction.

**Fig. 2:**
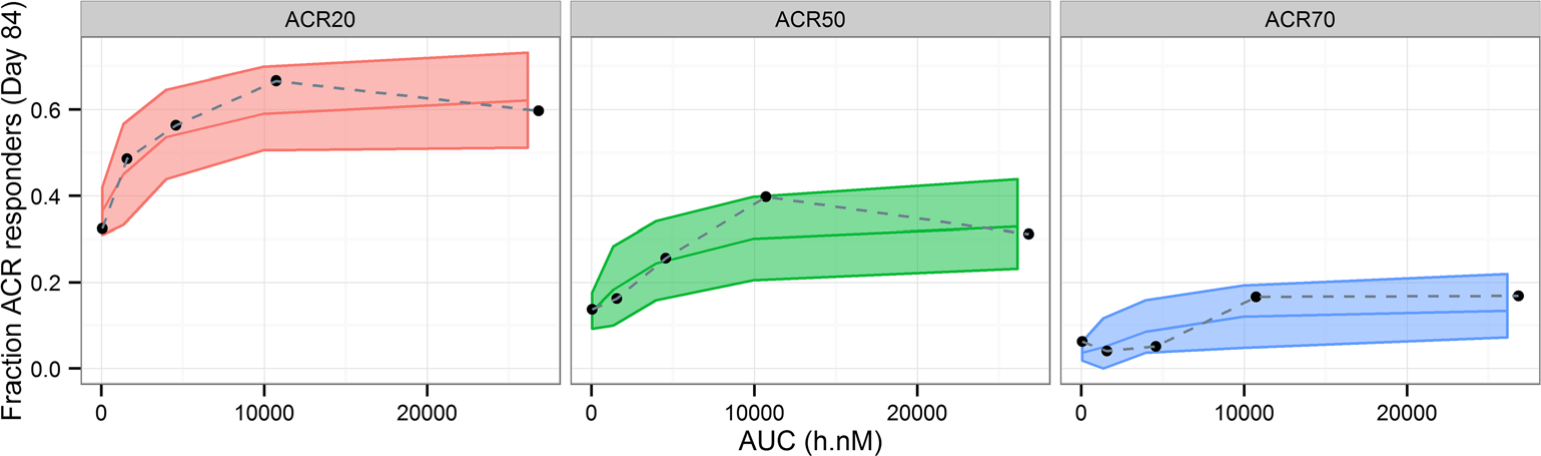
Posterior predictive check for exposure-response model of ACR20, ACR50, and ACR70 – by fenebrutinib AUC bins at week 12. AUC, area under the concentration time curve. Black dots: observed responder fraction at fenebrutinib exposure quartiles; bands: 90% prediction interval; red, green, blue middle lines: median prediction.

The final E-R model fit the data adequately as evident in the posterior predictive check (PPC) plots that assessed the performance of the models: (a) longitudinal probability of achieving ACR20, ACR50, and ACR70 stratified by fenebrutinib dose (Fig. 1**)**, and (b) fenebrutinib exposure effect (Fig. 2). The 90% prediction interval for the 50 mg QD dose group is wider than the other 3 dose groups, due to the smaller number of subjects from the 50 mg QD arm compared to the others in the Phase 2 trial. The correlation coefficients (r values) between ACR20 and ACR50, between ACR50 and ACR70, and between ACR20 and ACR70 are 0.415, 0.59, and 0.245, respectively, for observed data, and 0.439, 0.613, and 0.269, respectively, for model-based simulation.

#### DAS28 (CRP)

The longitudinal measurements of DAS28 (CRP) were described by a logistic model that includes the effects of treatments as a function of time, fenebrutinib exposure in terms of steady state AUC, and patient characteristics. The time component was incorporated as a sigmoidal E_max_ function to represent efficacy increases to a maximum effect over time, with separate parameters for fenebrutinib and placebo for the time at which each reached 50% of maximum effect.

Exploration of an E-R relationship between fenebrutinib exposure, in terms of C_max_, C_min_, and AUC at steady state, and DAS28 (CRP) levels was performed. As with the E-R analysis conducted using ACR response data, AUC was chosen as the exposure metric with which to conduct subsequent E-R analysis using DAS28 (CRP). The fenebrutinib exposure effect was modeled as a simple E_max_ function. Inter-individual variability was placed on baseline and the maximum effect parameters. The NONMEM control stream for the model and the parameter estimates are shown in Model S3 and Table S5, respectively.

**Fig. 3:**
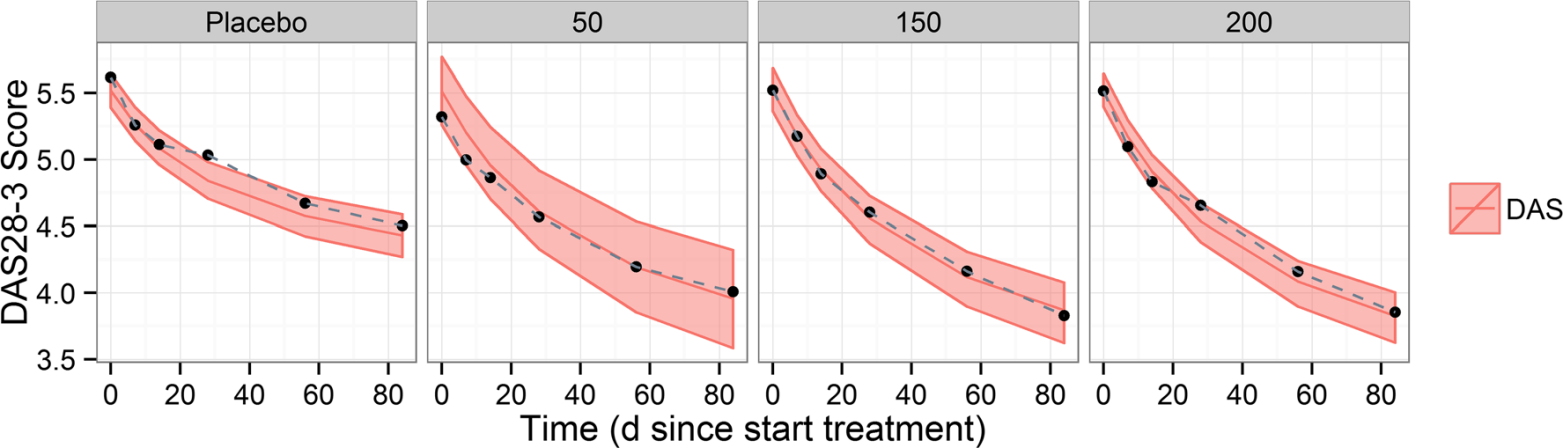
Visual predictive check of DAS28 (CRP) exposure-response model – time course by fenebrutinib dose. Black dots: observed responder fraction; bands: 90% prediction interval; red middle lines: median prediction.

**Fig. 4:**
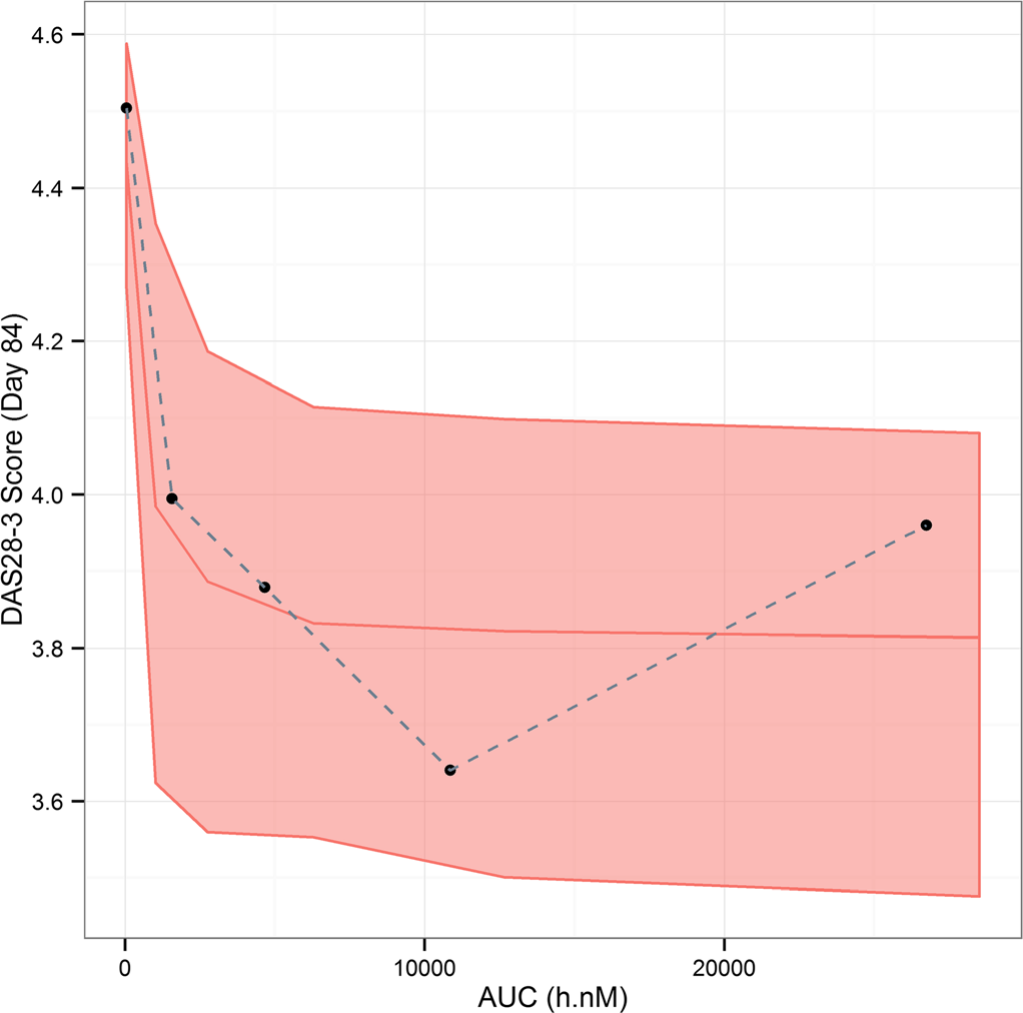
Visual predictive check of DAS28 (CRP) exposure-response model – by fenebrutinib AUC bins at week 12. AUC, area under the concentration time curve. Black dots: observed responder fraction at fenebrutinib exposure quartiles; bands: 90% prediction interval; red middle lines: median prediction.

The VPC plots (Fig. 3 for the timecourse of DAS28 (CRP) stratified by fenebrutinib dose; Fig. 4 for the effect of fenebrutinib exposure) were used to assess the performance of the models, and show that the final model fit the data adequately.

### Model-Based Meta-Analysis (MBMA) of ACR20, ACR50, and ACR70

The meta-analysis database contained publicly available trial data from 62 citations and included 9 drugs of multiple mechanisms of action plus placebo. Notably, among these were 16 citations providing summary metrics from 1871 patients for adalimumab, 51 providing summary metrics for placebo from 6567 patients, and 10 containing summary metrics from 1132 patients for tofacitinib, collectively used to characterize both the longitudinal course of effect over time, and dose-response for drugs of interest. For each treatment, there were between 1 and 6 dose levels (median of 3), and between 1 and 47 observation times (median of 11). All of the above metrics also applied to the ACR50 subset of the database, except that it contained between 1 and 44 observation times (median 10.5). Observations from fenebrutinib Phase 2 trial were summarized at the study level and added to the database. Table S6 summarize the ACR20, ACR50, and ACR70 data, and sources from the database.

Analysis of summary-level treatment efficacy from fenebrutinib and published RA trial data was described by a logistic model, using the observed fraction of patients who achieved the three ACR improvement thresholds. The model incorporated the effect of placebo or active treatment as a function of time, as well as the effects of drug doses, and patient population characteristics. The time component was modeled as an exponential function reflecting the growth to a maximum effect, and the dose-response varied by treatment. Where appropriate, an E_max_ function was used to describe the dose-response, while in others, a linear relationship or a single level of treatment effect was estimated. If data from different dosing schedules were available, the dose was normalized to total amount in one week in order to pool data for the same treatment across multiple trials, when necessary. Separate baselines were estimated for each of the ACR20, ACR50, and ACR70. The model included a function to ensure that the resulting predictions for ACR20, ACR50, and ACR70 were in proper order. Between-trial and between-arm variability was modeled on baseline, while between-drug variability was modeled on the rate of change in the time course.

The dose-response of fenebrutinib on ACR response endpoints in the MBMA model was informed by leveraging patient-level data described for E-R analysis. The dose at which 50% of efficacy (ED_*50*_ = 525 mg/week) in the MBMA was calculated and fixed in the model using the model-predicted exposure associated with 50% of efficacy (EC_*50*_ = 2650 ng*h/mL) that was estimated in the E-R analysis for ACR20, ACR50, and ACR70. Lastly, covariate analysis using forward addition found a high proportion (>80%) of patients who had inadequate response to previous anti-TNF therapy (TNF-IR), the percentage of patients who had failed previous MTX treatment (MTX-IR), and concurrent MTX therapy had statistically significant impact on ACR response rates. The R code for the final MBMA model is shown in Data S1.

**Fig. 5:**
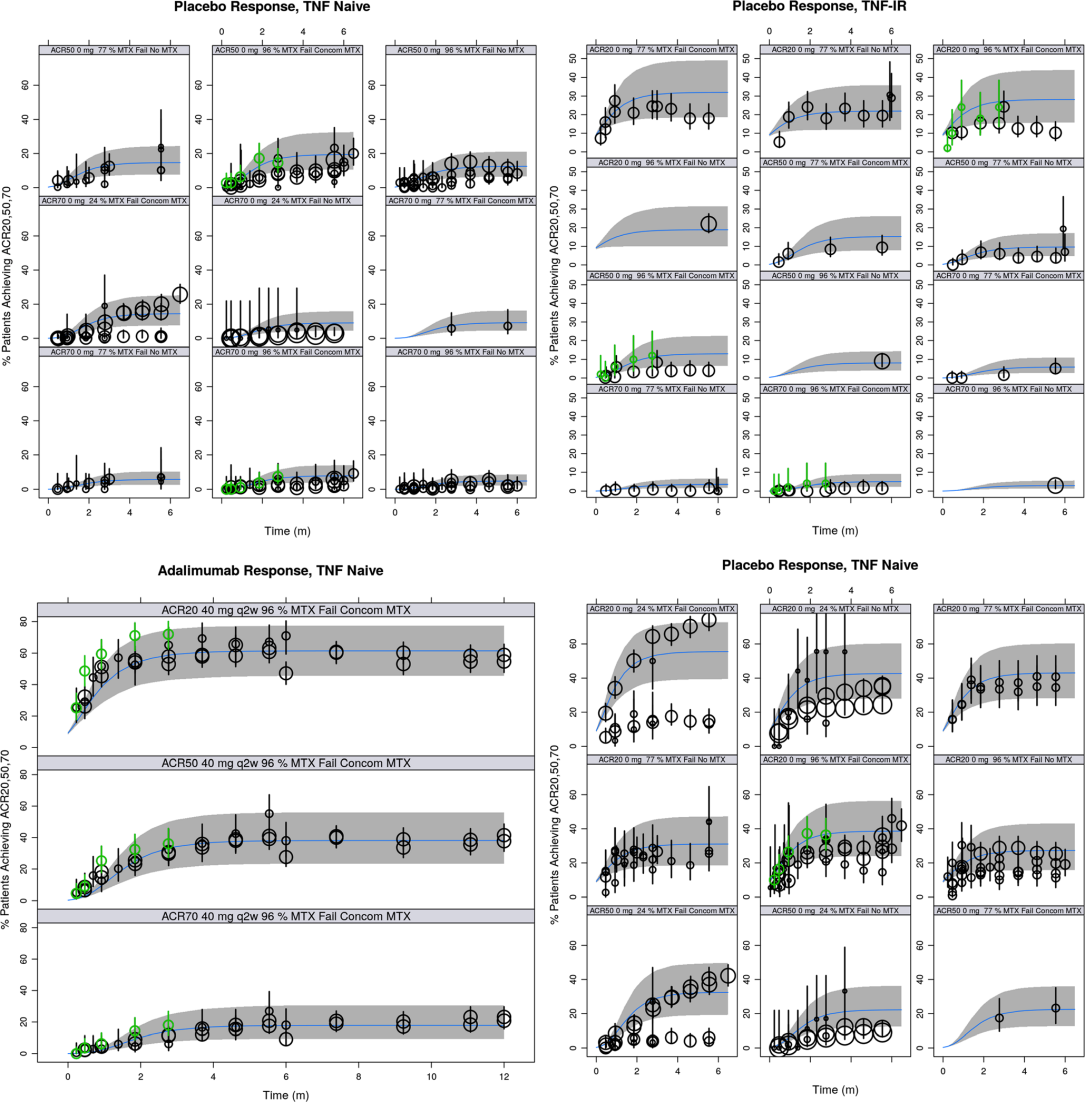
Posterior predictive check for model-based meta-analysis model - time course by endpoint, dose, and patient subgroup for placebo and adalimumab treatments. TNF, tumor necrosis factor; IR, inadequate response, MTX, methotrexate; q2w, once every two weeks; concom, concomitant. black dots: observed responder fraction with symbol size scaled to sample size; black lines: 90% confidence interval for observations; green dots and lines: observed summary-level data and 90% confidence interval from fenebrutinib phase 2 trial; bands: 90% prediction interval; blue middle line: median prediction.

**Fig. 6:**
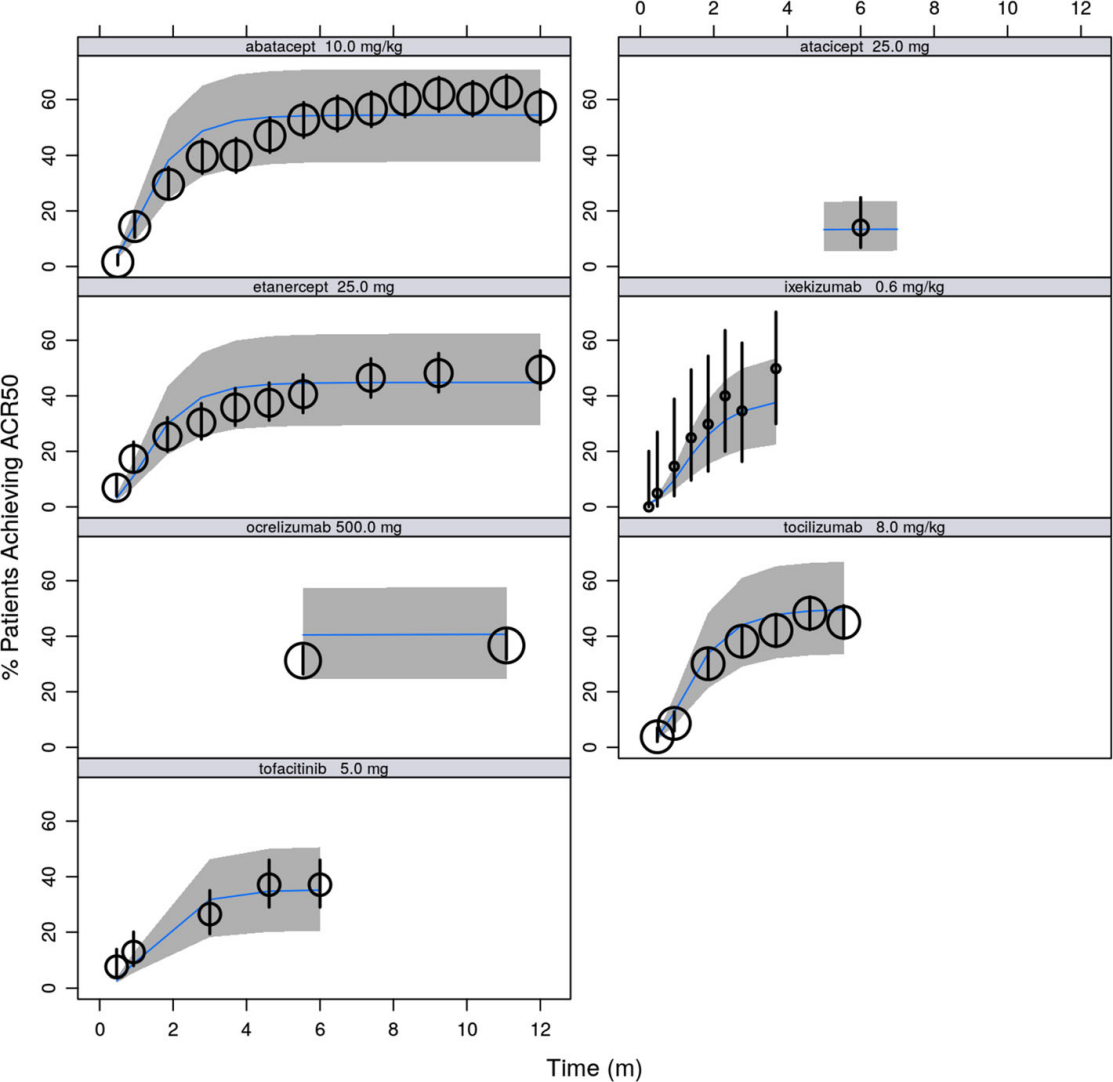
Posterior predictive check for model-based meta-analysis model - time course of ACR50 for other drugs leveraged (selected doses). Note: only one dose level per treatment is shown as representative example. Black dots: observed responder fraction with symbol size scaled to sample size; black lines: 90% confidence interval for observations; bands: 90% prediction interval; blue middle line: median prediction.

The ACR20, ACR50, and ACR70 response rates in the placebo and adalimumab arms of the fenebrutinib Phase 2 trial were found to be consistent with historical data for these treatments (Fig. 5). Additionally, the model performance was validated using observed response rates across the treatments used, their doses, and the longitudinal time courses of effect against 90% prediction intervals for each treatment, as seen in representative treatments in Fig. 6.

Leveraging data across all 10 treatments in the model, the final MBMA model estimates that populations with at least 80% of patients who are TNF-IR would have lower response than those with higher percentages of patients who had previously failed on MTX. Background use of MTX resulted in increased patient population response rates.

**Fig. 7:**
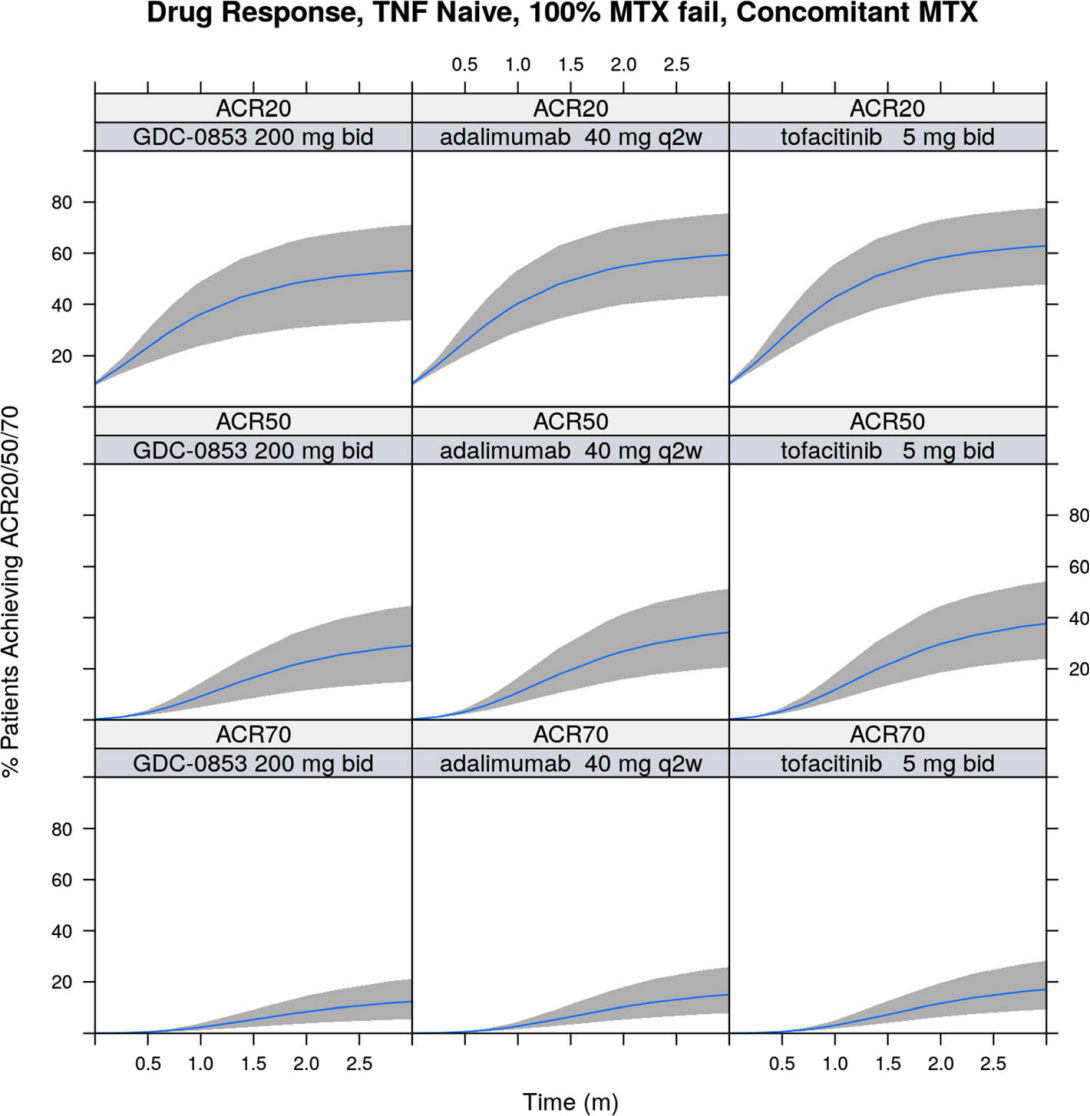
ACR20, ACR50, and ACR70 simulation for fenebrutinib (GDC-0853), adalimumab, and tofacitinib treatments in TNF-inhibitor-naïve Population. TNF, tumor necrosis factor; MTX, methotrexate; bid, twice daily; q2w, once every two weeks. Bands: 90% prediction interval; blue middle line: median prediction.

The utilization of comprehensive historical data through MBMA enabled comparison of fenebrutinib *versus* its potential comparator compounds. Simulation based on the developed MBMA model showed that 200 mg BID fenebrutinib was predicted to have similar efficacy in terms of ACR20, ACR50, and ACR70 compared to the registrational doses of adalimumab and tofacitinib in a MTX-IR (but anti-TNF-naïve) population at 12 weeks after initiation of treatment, when incorporating variability in PK and efficacy endpoints, as depicted in Fig. 7. Simulation results for TNF-IR patient populations are included in Fig. S4 to demonstrate, as an example, the utility of applying the developed MBMA model to predict efficacy in another patient population, which showed that 200 mg BID fenebrutinib would have comparable efficacy to the registrational doses of adalimumab and tofacitinib in terms of ACR20, ACR50, and ACR70, in TNF-IR patients at 12 weeks after initiation of treatment.

## Discussion

The wealth and diversity of efficacy data among various treatments in RA clinical trials necessitate an integrated approach to fully leverage knowledge from publicly available summary-level data and understand the impact of treatments based on new mechanisms of action. Therefore, we aimed to design a novel study with multiple model-based analyses using data from the fenebrutinib Phase 2 trial in patients with RA, which we report here.

### Population Pharmacokinetics (popPK) Analysis

The popPK analysis conducted herein provided a useful representation of the exposures resulting from investigated doses of fenebrutinib, and the effects of patient characteristics on the inter-subject variability of the PK parameters. Food and PPI effects were incorporated into the model using TCAM, because previous knowledge using non-compartmental analysis from the Phase 1 relative bioavailability/food effect/drug-drug interaction study indicated that food blunts the concentration-time profile and delays the T_max_, whereas PPI decreases the C_max_. In addition, out of the other statistically significant covariates in the final popPK model, covariate exploration found that the age and subject status in the analysis dataset could be confounded; i.e. healthy subjects tend to be younger than patients with RA. Shrinkage values for interindividual variability and residual error terms in the popPK model are within an acceptable range, and along with the results from EBE-based model diagnostic methods, these indicate the observed data provided sufficient information for individual posthoc exposures estimation for the E-R analyses. Finally, if a larger sample size were available, additional causes of inter-subject variability in patients could be further investigated.

### Exposure-Response (E-R) Analyses

The primary goals of the E-R analyses with fenebrutinib were to characterize the relationship between exposure and clinical efficacy endpoints of ACR responses and DAS28 (CRP) levels, and to determine whether the efficacy could be further augmented with increasing exposure or dose. The longitudinal data of both ACR responses and DAS28 (CRP) levels were modeled. Graphical exploration and model-based simulation suggest that a plateau of efficacy was achieved within the exposure range obtained in the study, and further increase in fenebrutinib dose would not lead to additional efficacy in cohort 1 patients who were MTX-IR. The lack of difference in statistical significance between C_max_, C_min_, and AUC as the driver for E-R is likely due to the high correlation between the three exposure metrics in the Phase 2 trial. Though region was the only covariate in the longitudinal E-R model, region and baseline RF were correlated in the patient population evaluated (i.e. Latin America had higher baseline RF values). Therefore, it might be difficult to disentangle whether the covariate effect was truly due to region or other regional differences, such as baseline RF levels, which are important in the diagnosis and determination of RA severity (35). Interestingly, treatment history (MTX-IR *vs.* TNF-IR) was tested but was not a significant covariate in the E-R model, indicating similar E-R between MTX-IR and TNF-IR patients. This could possibly be due to the difference in background treatment or disease management between the two populations in this study. Ultimately, E-R in cohort 2 patients, who were TNF-IR, is unclear due to the small sample size and limited PK exposure range investigated.

### Model-Based Meta-Analysis (MBMA)

Modeling of historical meta-analysis data sought to leverage publicly available information to enable direct and indirect comparisons against competitors and between study populations. Such an analysis is particularly valuable at the end of Phase 2 milestone for informing go/no-go decisions to proceed to Phase 3 (36). First, MBMA aimed to quantitatively compare fenebrutinib against currently marketed therapies, through an indirect comparison of the performance of placebo and adalimumab historically *versus* that observed in the fenebrutinib Phase 2 trial. Second, MBMA sought to better inform the magnitude of impact of patient population covariates, with the initial interest focusing on informing the impact of having patients who were MTX-IR *versus* other patient populations, if possible.

Efficacy benchmarking against approved agents was focused on adalimumab and tofacitinib within this body of work, however, relative performance of other agents could also be compared using the established MBMA model. Tofacitinib is a janus kinase (JAK) inhibitor previously approved for the treatment in patients with an inadequate response to MTX, which was the target population in cohort 1 of the fenebrutinib Phase 2 trial as well. Further, covariates in the model could be used for clinical trial simulations in populations not yet investigated, permitting future decision makers to consider the merits of new or altered therapies far before committing large investments.

There was also a useful transfer of information from the E-R analysis to the MBMA model development. Initially, it was difficult to obtain a stable estimate of the ED_*50*_ of fenebrutinib in the MBMA model. However, by leveraging the estimated EC_*50*_ from the E-R analysis, which is based on data from individual subjects, the final MBMA model that is based on summary-level data is much more informed in regard to the treatment effect of fenebrutinib.

The analysis had some limitations. Notably, efficacy of fenebrutinib beyond 12 weeks is unknown, limiting comparison at such observation times. Although MBMA analysis by *Wang et al* showed that ACR50 responses at 3 months after initiation of treatment is predictive of long term efficacy for most drug classes (37), this assumption might not apply to a molecule with a new mechanism of action and possibly a slower onset of action, and therefore longer monitoring to accurately determine maximum efficacy could be warranted. Further, the MBMA had slightly different prediction results than observed (non-modeling) data from the fenebrutinib Phase 2 trial (24). It is hypothesized that the study patients from all arms are slightly over performing compared to median historical results for placebo and adalimumab treatments, although still within the variability of historical data. In our experience, MBMA can often yield slightly different results for an individual trial when it is modeled along with historical data, given the weight and richness of information from the latter.

## Conclusions

The multiple analysis types as described herein provided an integrated view of model-based evidence, which has not been reported previously for a BTK inhibitor in patients with RA. This holistic approach yielded more insight than separate analyses alone, especially at an earlier phase of the drug development, where the uncertainly of treatment effect estimation and prediction could be large. Thus, the approach examines the totality of available evidence and allows better informed decision-making, and therefore is consistent with model-based strategies that are currently being evaluated by PDUFA VI to support drug development.

## Electronic supplementary material

ESM 1 (DOCX 938 kb)

## Abbreviations

AUC: Area under the concentration-time curve
BTK: Bruton’s tyrosine kinase
CL/F: Apparent clearance
C_max_: Maximum concentration
C_min_: Trough concentration
CRP: C-reactive protein
DAS28: Disease Activity Score with 28-joint counts
EBE: Empirical Bayes estimates
E-R: Exposure-response
F1: Relative extent of absorption
FOCE-I: First order conditional estimation with interaction
MBMA: Model-based meta-analysis
MIDD: Model-informed drug development
MTT: Mean transit time
MTX: Methotrexate
NLME: Non-linear mixed effects
NTR: Number of transit compartments
pcVPC: Prediction-corrected visual predictive check
PD: Pharmacodynamic
PK: Pharmacokinetics
popPK: Population pharmacokinetics
PPC: Posterior predictive check
PPI: Proton pump inhibitor
RA: Rheumatoid arthritis
RF: Rheumatoid factor
SLE: Systemic lupus erythematosus
TCAM: Transit compartment absorption model
T_max_: Time associated with maximum concentration
VPC: Visual predictive check
