# Supplementary material for: Population Pharmacokinetics, Efficacy Exposure-response Analysis, and Model-based Meta-analysis of Fenebrutinib in Subjects with Rheumatoid Arthritis
Source: Pharm Res. 2020 Jan 6;37(2):25. doi: 10.1007/s11095-019-2752-y (PMC6944649; doi:10.1007/s11095-019-2752-y)
Supplement: Supplementary file 1 — (DOCX 938 kb) [file 11095_2019_2752_MOESM1_ESM.docx]

**Population pharmacokinetics, efficacy exposure-response analysis, and model-based meta-analysis of fenebrutinib in subjects with rheumatoid arthritis**

Phyllis Chan, PhD^1^, Jiajie Yu, PhD^1^, Leslie Chinn, PhD^1^, Marita Prohn, MSc^2^, Jan Huisman, BEng^2^, Brett Matzuka, PhD^3^, William Hanley, PhD^4^, Katie Tuckwell, PhD^5^, Angelica Quartino, PhD^1^

^1^Department of Clinical Pharmacology, Genentech, South San Francisco, CA, USA

^2^qPharmetra, Nijmegen, The Netherlands

^3^qPharmetra, Cary, NC, USA

^4^Former Genentech employee, currently of Seattle Genetics, South San Francisco, CA, USA

^5^Early Clinical Development, Genentech, South San Francisco, CA, USA

**Supplementary Figures and Tables:**

**Table S1.** Summary of Studies Included in the Population PK Analysis

**Table S2.** Summary of Demographics of Subjects Included in the Population PK Analysis

**Table S3.** Summary of Demographics of Subjects Included in the E-R Analysis

**Table S4.** ACR20, ACR50, ACR70 E-R Model Parameter Estimates

**Table S5.** DAS28-3 CRP E-R Model Parameter Estimates

**Table S6.** Summary of treatments and clinical trials in the model-based meta-analysis dataset

**Figure S1.** Diagnostic Plots of Population PK Model

**Figure S2.** Prediction-Corrected Visual Predictive Check Plots of Population PK Model

**Figure S3.** Distribution of Fenebrutinib Exposure by Dose

**Figure S4.** ACR Simulation for Fenebrutinib (GDC-0853), Adalimumab, and Tofacitinib Treatments

**Model S1.** NONMEM Control Stream for Population PK Model

**Model S2.** NONMEM Control Stream for ACR E-R Model

**Model S3.** NONMEM Control Stream for DAS28 E-R Model

**Data S1.** R Code for MBMA Model

**Table S1 Summary of Studies Included in the Population PK Analysis**

| **Study Number** | **Study Description** | **Trial Design** | **Fenebrutinib Pharmacokinetic Sampling Schedule** |
| --- | --- | --- | --- |
| GA29347 | Phase 1 multiple ascending dose in healthy volunteers | Subjects received the powder-in-capsule formulation of fenebrutinib at 20 mg BID, 60 mg BID, 150 mg BID, 250 mg BID, or 500 mg QD for 14 days. | Pre-dose on days 1, 2, 3, 5, 7, and 14, and post-dose (morning dose) on days 1 and 14 at 0.5, 1, 2, 3, 4, 6, 8, 11.75 13, 14, and 16 hours, as well as after the last dose on day 14 at 24, 36, and 48 hours. For BID dosing, additional sampling on days 1 and 14 at 13, 14, and 16 hours after the morning dose. |
| GP29832 | Phase 1 relative bioavailability/food effect/drug-drug interaction in healthy volunteers | In Part 1, subjects received 200 mg fenebrutinib in a crossover design as either the powder-in-capsule or tablet formulation in the fasted state (Treatment A or B) with the final fixed treatment consisting of the tablet formulation administered in the fasted state after prior administration of rabeprazole for 3 days (Treatment C).  In Part 2, subjects received 200 mg fenebrutinib of the tablet formulation in the fasted or fed state (Treatment D or E) in a crossover design with the final fixed treatment consisting of the tablet formulation administered in the fed state after prior administration of rabeprazole for 3 days (Treatment F).  In Part 3, subjects received a single low-dose (7.5 mg) of methotrexate in the fasted state (Treatment G) followed by a washout period of 13 days. Subsequently, subjects received six days of twice-daily administration of the GDC-0853 tablet formulation (Treatment H), with the final treatment consisting of a single low-dose (7.5 mg) of methotrexate administered together with a single dose of GDC-0853 (Treatment I). The fenebrutinib dose administered in Part 3 was 200 mg BID for Treatment H and a single 200 mg dose for Treatment I. | In Parts 1 and 2: pre-dose, and 0.5, 1, 2, 3, 4, 6, 8, 10, 12, 24, 36, 48, and 72 hours post-dose.  In Part 3: pre-dose on days 17, 18, 19, 20, and 21; post-dose on days 20 and 21 at 0.5, 1, 2, 3, 4, 6, 8, 10, 12 hours, and on day 21 at 16 and 24 hours. |
| GA29350 | Phase 2 in patients with rheumatoid arthritis | In cohort 1, patients were assigned to 1 of 5 parallel treatment arms (3 fenebrutinib arms, 1 placebo arm, and 1 adalimumab arm). In the fenebrutinib and placebo arms, subjects received 50 mg QD, 150 mg QD, or 200 mg BID fenebrutinib tablets or placebo tablets, together with subcutaneous (SC) placebo injections every other week for 12 weeks (n=40 in the 50 mg arm and n=109 to 111 in each of the other 4 arms). In the active comparator arm, patients received adalimumab 40 mg SC every other week and placebo tablets for 12 weeks. In cohort 2 (n=98 enrolled), patients received 200 mg fenebrutinib BID or placebo tablets for 12 weeks. Food co-administration was self-reported by the patients. | Pre-dose (morning dose) on days 1, 7, 28, 56, and 84 (all patients) and at 2 hours, 4-6 hours, and 8-10 hours (US sites only, N=8) after dosing (morning dose) on day 28 |

**Table S2 Summary of Demographics of Subjects Included in the Population PK Analysis**

|  | **GP29832** | **GA29347** | **GA29350** | **All** |
| --- | --- | --- | --- | --- |
|  | **(N=48)** | **(N=30)** | **(N=307)** | **(N=385)** |
|  |  |  |  |  |
| **Gender** |  |  |  |  |
| Female | 1 (2.1%) | 5 (16.7%) | 249 (81.1%) | 255 (66.2%) |
| Male | 47 (97.9%) | 25 (83.3%) | 58 (18.9%) | 130 (33.8%) |
|  |  |  |  |  |
| **Age (yr)** |  |  |  |  |
| Mean (SD) | 31.6 (11) | 37.1 (7.5) | 50.6 (12) | 47.2 (13) |
| Median (range) | 28 (18 - 55) | 35 (25 - 51) | 52 (19 - 75) | 48 (18 - 75) |
|  |  |  |  |  |
| **Weight (kg)** |  |  |  |  |
| Mean (SD) | 81.5 (9.6) | 80.9 (14) | 72.9 (16) | 74.6 (16) |
| Median (range) | 81 (60.8 - 103) | 80.2 (55.4 - 102) | 71 (38 - 153) | 73 (38 - 153) |
|  |  |  |  |  |
| **BMI (kg/m2)** |  |  |  |  |
| Mean (SD) | 26 (2.9) | 26.5 (2.9) | 27 (5.7) | 26.8 (5.3) |
| Median (range) | 26.2 (20.2 - 30.5) | 27.1 (20.5 - 30.7) | 26.1 (16.3 - 61.3) | 26.2 (16.3 - 61.3) |
|  |  |  |  |  |
| **C-reactive Protein (mg/L)** |  |  |  |  |
| Mean (SD) | 1.08 (1.7) | not available | 21.5 (25) | 17.3 (24) |
| Median (range) | 0.7 (0 - 8.8) | not available | 12 (0.3 - 176) | 9.2 (0 - 176) |
|  |  |  |  |  |
| **ALT (U/L)** |  |  |  |  |
| Mean (SD) | 21.2 (8.8) | not available | 18.8 (10) | 19.1 (9.8) |
| Median (range) | 19 (11 - 69) | not available | 16 (6 - 71) | 18 (6 - 71) |
|  |  |  |  |  |
| **AST (U/L)** |  |  |  |  |
| Mean (SD) | 20.7 (3.4) | not available | 18.6 (8.3) | 19 (7.5) |
| Median (range) | 20 (16 - 38) | not available | 17 (6 - 100) | 18 (6 - 100) |
|  |  |  |  |  |
| **Creatinine Clearance (mL/min)** |  |  |  |  |
| Mean (SD) | 114 (11) | not available | 118 (37) | 117 (34) |
| Median (range) | 111 (101 - 156) | not available | 112 (47.3 - 251) | 111 (47.3 - 251) |
|  |  |  |  |  |
| **Total Protein (g/L)** |  |  |  |  |
| Mean (SD) | 71 (2.8) | not available | 72.6 (5.4) | 59 (46) |
| Median (range) | 71 (65 - 78) | not available | 73 (58 - 94) | 71 (-99 - 94) |
|  |  |  |  |  |
| **Race** |  |  |  |  |
| White | 0 (0.0%) | 14 (46.7%) | 269 (87.6%) | 283 (73.5%) |
| Black | 0 (0.0%) | 15 (50.0%) | 4 (1.3%) | 19 (4.9%) |
| Asian | 0 (0.0%) | 0 (0.0%) | 1 (0.3%) | 1 (0.3%) |
| Am. Indian or Alaska Native | 0 (0.0%) | 0 (0.0%) | 26 (8.5%) | 26 (6.8%) |
| Multiple | 0 (0.0%) | 0 (0.0%) | 5 (1.6%) | 5 (1.3%) |
| Missing | 48 (100.0%) | 1 (3.3%) | 2 (0.7%) | 51 (13.2%) |
|  |  |  |  |  |
| **Subject Status** |  |  |  |  |
| Healthy volunteer | 48 (100.0%) | 30 (100.0%) | 0 (0.0%) | 78 (20.3%) |
| Patient | 0 (0.0%) | 0 (0.0%) | 307 (100.0%) | 307 (79.7%) |
|  |  |  |  |  |
| **Food** |  |  |  |  |
| Fasted | 40 (83.3%) | 30 (100.0%) | 2 (0.7%) | 72 (18.7%) |
| Fed | 8 (16.7%) | 0 (0.0%) | 305 (99.3%) | 313 (81.3%) |
|  |  |  |  |  |
| **PPI Comedicated** |  |  |  |  |
| No | 48 (100.0%) | 30 (100.0%) | 185 (60.3%) | 263 (68.3%) |
| Yes | 0 (0.0%) | 0 (0.0%) | 122 (39.7%) | 122 (31.7%) |
|  |  |  |  |  |
| **MTX-IR** |  |  |  |  |
| Not applicable | 48 (100.0%) | 30 (100.0%) | 0 (0.0%) | 78 (20.3%) |
| Yes | 0 (0.0%) | 0 (0.0%) | 307 (100.0%) | 307 (79.7%) |
|  |  |  |  |  |
| **TNF-IR** |  |  |  |  |
| No | 0 (0.0%) | 0 (0.0%) | 259 (84.4%) | 259 (67.3%) |
| Not applicable | 48 (100.0%) | 30 (100.0%) | 0 (0.0%) | 78 (20.3%) |
| Yes | 0 (0.0%) | 0 (0.0%) | 48 (15.6%) | 48 (12.5%) |

PK, pharmacokinetic; E-R, exposure-response; N, number of subjects; SD, standard deviation; BMI, body mass index; Am., American; ALT, alanine aminotransferase; AST, aspartate aminotransferase; PPI, proton pump inhibitor; MTX, methotrexate; TNF, tumor necrosis factor; IR, inadequate response.

**Table S3 Summary of Demographics of Subjects Included in the E-R Analysis**

|  | **Mean** | **SD** | **Median** | **Range (Min, Max)** | **Missing** |
| --- | --- | --- | --- | --- | --- |
| **Rheumatoid Factor (IU/mL)** | 323.8 | 489.2 | 124.0 | (14.0 - 3,060.0) | 2 |
|  |  |  |  |  |  |
| **Disease Duration at Baseline (d)** | 2,837 | 2,538 | 2,122 | (51 - 13,945) | 6 |
|  |  |  |  |  |  |
| **Creatinine Clearance (mL/min)** | 117.3 | 37.7 | 111.0 | (47.3 - 323.8) | 1 |
|  |  |  |  |  |  |
| **Age (y)** | 50.9 | 11.9 | 52.0 | (19.0 - 75.0) | 0 |
|  |  |  |  |  |  |
| **Weight (kg)** | 73.1 | 16.3 | 71.0 | (38.0 - 153.0) | 1 |
|  |  |  |  |  |  |
| **C-Reactive Protein (mg/L)** | 22.4 | 26.4 | 12.2 | (0.3 - 181.0) | 11 |
|  |  |  |  |  |  |
| **Categorical Covariates** (N=467) |  |  |  |  |  |
| **Region** |  | | | | |
| US | 22 (5%) | | | | |
| Eastern Europe | 290 (62%) | | | | |
| Latin America | 155 (33%) | | | | |
| **Smoking Status** |  | | | | |
| Current | 109 (23%) | | | | |
| Never | 358 (77%) | | | | |
| **Prior MTX-IR** |  | | | | |
| No | 0 (0%) | | | | |
| Yes | 467 (100%) | | | | |
| **Prior anti-TNF-IR** |  | | | | |
| No | 369 (79%) | | | | |
| Yes | 98 (21%) | | | | |
| **Prior Biologics Treatment** |  | | | | |
| No | 369 (79%) | | | | |
| Yes | 98 (21%) | | | | |
| **Gender** |  | | | | |
| Female | 376 (81%) | | | | |
| Male | 91 (19%) | | | | |

E-R, exposure-response; N, number of subjects; SD, standard deviation; MTX, methotrexate; TNF,

tumor necrosis factor; IR, inadequate response.

**Table S4. ACR20, ACR50, ACR70 E-R Model Parameter Estimates**

| **Parameter** | **Alias** | **Estimate** | **Relative SE (%)** | **95% CI** | **Shrinkage (%)** |
| --- | --- | --- | --- | --- | --- |
| θ1 | ACR20 baseline | -4.62 | 10 | (-5.52 - -3.72) |  |
| θ2 | ACR50 baseline | -6.47 | 6.4 | (-7.29 - -5.66) |  |
| θ3 | ACR70 baseline | -8.3 | 5.7 | (-9.22 - -7.38) |  |
| θ4 | Markov component | 0.934 | 17 | (0.623 - 1.25) |  |
| θ7 | Maximum placebo effect over time | 3.41 | 14.5 | (2.44 - 4.38) |  |
| θ8 | Time of 50% placebo effect - ACR20 (d) | 21.5 |  | (16.6 - 27.7) |  |
| θ9 | Max drug effect over time - Eastern Europe | 1.39 | 29.8 | (0.578 - 2.21) |  |
| θ10 | Exposure at which 50% drug effect (AUC ng∙hr /mL) | 2650 |  | (675 - 10400) |  |
| θ11 | Time of 50% placebo effect - ACR50 and ACR70 (d) | 32.8 |  | (26.1 - 41.1) |  |
| θ12 | Hill coefficient on time course | 2.52 |  | (1.5 - 4.23) |  |
| θ13 | Max drug effect over time - US | 2.13 | 39.7 | (0.472 - 3.78) |  |
| θ14 | Max drug effect over time - Latin America | 2.06 | 25.8 | (1.02 - 3.1) |  |
| ω16.1 | ω2IOV on baseline | 4.85 | 13.3 | (3.59 - 6.11) | 20.45 |

**Table S5. DAS28-3 CRP E-R Model Parameter Estimates**

| **Parameter** | **Alias** | **Estimate** | **Relative SE (%)** | **95% CI** | **Shrinkage (%)** |
| --- | --- | --- | --- | --- | --- |
| θ1 | Baseline DAS28 score | 5.46 | 0.9 | (5.37 - 5.55) |  |
| θ2 | Maximum placebo effect over time (DAS28) | -1.36 | 7.9 | (-1.57 - -1.15) |  |
| θ3 | Time of 50% placebo effect (d) | 36.7 |  | (25.7 - 52.4) |  |
| θ4 | Time of 50% drug effect (d) | 47 |  | (39.2 - 56.3) |  |
| θ5 | Exposure at which 50% drug effect (AUC ng∙hr/mL) | 293 |  | (14.5 - 5920) |  |
| θ6 | Maximum drug effect over time (DAS28) | -0.964 | 19.4 | (-1.33 - -0.598) |  |
| ω1.1 | ω2Baseline | 0.0206 | 8.6 | (0.0171 - 0.0241) | 8.63 |
| ω2.2 | ω2Max drug effect | 0.283 | 13.6 | (0.208 - 0.358) | 25.06 |
| σ | Additive residual error in patients (%) | 0.3 | 2.2 | (0.287 - 0.313) |  |

| **Table S6: Summary of treatments and clinical trials in the model-based meta-analysis dataset**   \| **Treatment** \| **Dose** \| **Total number of arms** \| **Total number of timepoints** \| **Total number of subjects** \| **References: Last name of the first author (publication year)** \| \| --- \| --- \| --- \| --- \| --- \| --- \| \| Abatacept \| 2 mg/kg, 10 mg/kg, 125 mg \| 6 \| 13 \| 2125 \| Genovese (2005), Genovese (2011), Schiff (2008), Weinblatt (2007), Westhovens (2009) \| \| Adalimumab \| 1 mg/kg, 20 mg, 40 mg, 80 mg \| 23 \| 13 \| 2605 \| Breedveld (2006), Furst (2003), Roche/Genentech (2017), Genovese (2007), Kanik (2009), Keystone (2004), Mease (2005), Miyasaka (2008), Rau (2004), Soubrier (2009), van de Putte (2003), van de Putte (2004), van Vollenhoven (2011), Weinblatt (2003) \| \| Atacicept \| 25 mg, 75 mg, 150 mg \| 4 \| 1 \| 270 \| Genovese (2011), van Vollenhoven (2011) \| \| Etanercept \| 0.25 mg/m^2^, 2 mg/m^2^, 25 mg/m^2^, 10 mg, 25 mg, 50 mg \| 25 \| 13 \| 3396 \| Bathon (2000), Emery (2008), Genovese (2004), Johnsen (2006), Kameda (2010), Kameda (2011), Keystone (2004), Kim (2012), Klareskog (2004), Lan (2004), Mease (2000), Mease (2004), Moreland (1997), Moreland (1999), Sterry (2010), van der Heijde (2006), van der Heijde (2007), van Riel (2006), Weinblatt (1999), Weinblatt (2008) \| \| Fenebrutinib \| 50 mg, 150 mg, 200 mg \| 3 \| 4 \| 259 \| Roche/Genentech (2017) \| \| Ixekizumab \| 0.2 mg/kg, 0.6 mg/kg, 2.0 mg/kg \| 3 \| 5 \| 60 \| Genovese (2010) \| \| Ocrelizumab \| 200 mg, 500 mg \| 6 \| 3 \| 1644 \| Rigby (2012), Stohl (2012), Tak (2012) \| \| Placebo \| Not applicable \| 49 \| 15 \| 6039 \| Blanco (2017), Breedveld (2006), Burmester (2011), Burmester (2013), den Broeder (2002), Emery (2008), Fleischmann (2009), Fleischmann (2012), Furst (2003), Roche/Genentech (2017), Genovese (2005), Genovese (2007), Genovese (2008), Genovese (2010), Genovese (2011), Kanik (2009), Keystone (2004), Klareskog (2004), Kremer (2012), Kremer (2009), Kremer (2011), Kremer (2009), Lan (2004), Mease (2000), Mease (2004), Mease (2005), Miyasaka (2008), Moreland (1997), Moreland (1999), Rau (2004), Rigby (2012), Schiff (2008), Smolen (2008), Soubrier (2009), Stohl (2012), Tak (2012), Taylor (2015), van de Putte (2003), van de Putte (2004), van der Heijde (2006), van der Heijde (2007), van Vollenhoven (2011), Weinblatt (1999), Weinblatt (2003), Weinblatt (2007), Weisman (2003), Westhovens (2009), Yazici (2012) \| \| Tocilizumab \| 4 mg/kg, 8 mg/kg \| 8 \| 7 \| 2525 \| Dougados (2011), Emery (2008), Genovese (2008), Jones (2010), Smolen (2008), Yazici (2012) \| \| Tofacitinib \| 1 mg, 3 mg, 5 mg, 10 mg, 15 mg, 20 mg \| 39 \| 7 \| 3232 \| Burmester (2011), Burmester (2013), Fleischmann (2009), Fleischmann (2012), Kanik (2009), Kremer (2009), Kremer (2012), Kremer (2011), van Vollenhoven (2011) \| |  |  |  |  |  |  |  |
| --- | --- | --- | --- | --- | --- | --- | --- | --- | --- | --- | --- | --- | --- | --- | --- | --- | --- | --- | --- | --- | --- | --- | --- | --- | --- | --- | --- | --- | --- | --- | --- | --- | --- | --- | --- | --- | --- | --- | --- | --- | --- | --- | --- | --- | --- | --- | --- | --- | --- | --- | --- | --- | --- | --- | --- | --- | --- | --- | --- | --- | --- | --- | --- | --- | --- | --- | --- | --- | --- | --- | --- | --- | --- |

**Figures:**

**Figure S1 Diagnostic Plots of Population PK Model**


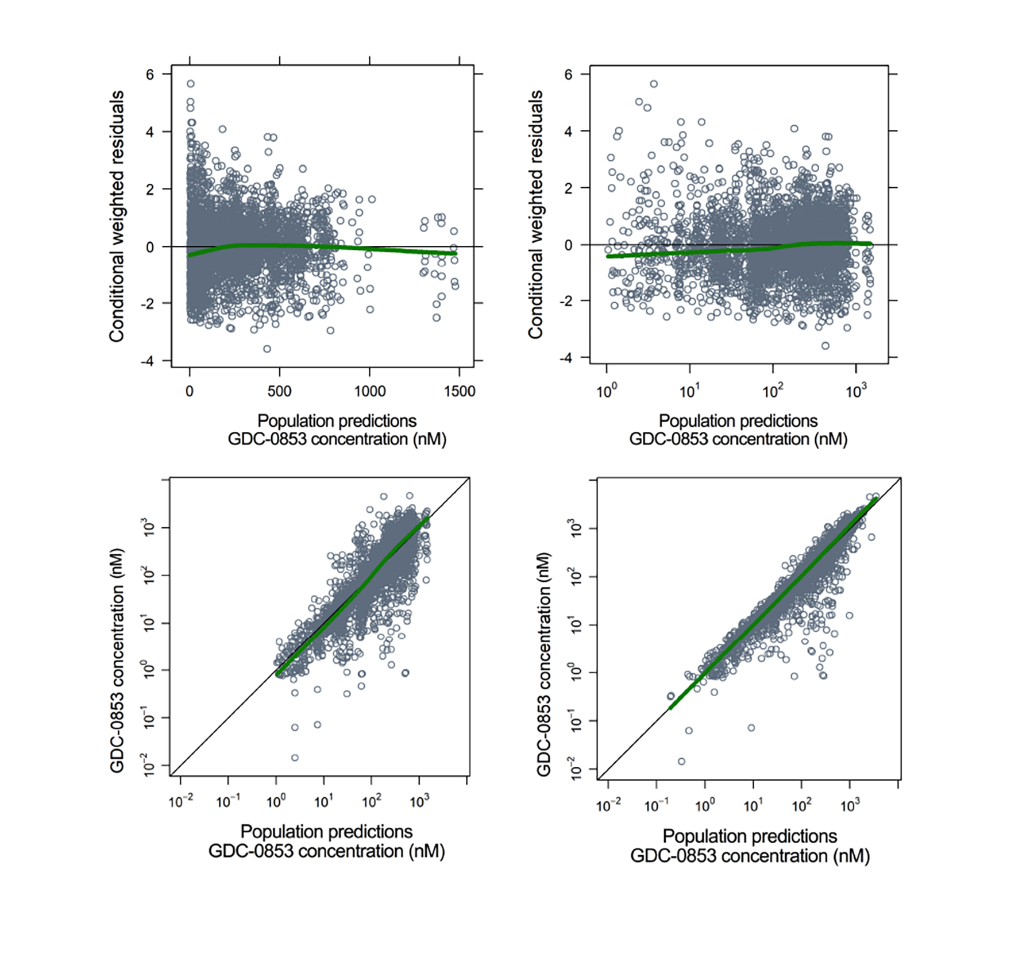


Green line: Loess smooth. Black line: Line of Identity. Open circles: observed data.

**Figure S2 Prediction-Corrected Visual Predictive Check Plot of Population PK Model**


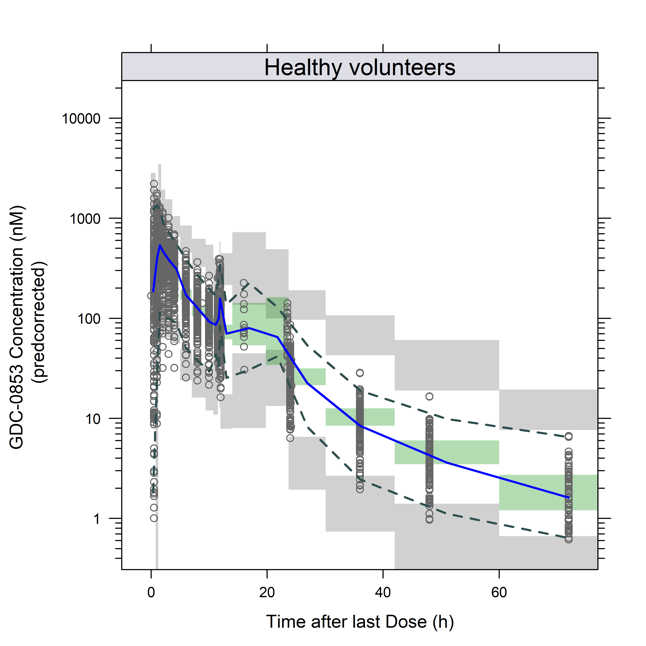

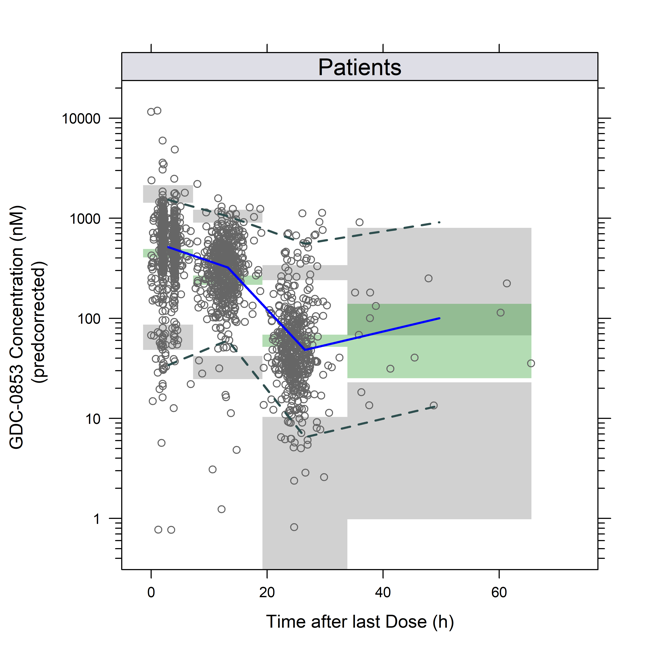


Circles: Observations, Solid Blue Line: Median of the observed fenebrutinib concentrations, Dashed Lines: 2.5th and 97.5th percentiles of the observed fenebrutinib concentrations, Shaded Area: The shaded areas indicate the 95% CI around the prediction-corrected median (green area), and 2.5th and 97.5th percentiles of the simulated concentrations (grey areas).

**Figure S3 Distribution of Fenebrutinib Exposure by Dose**


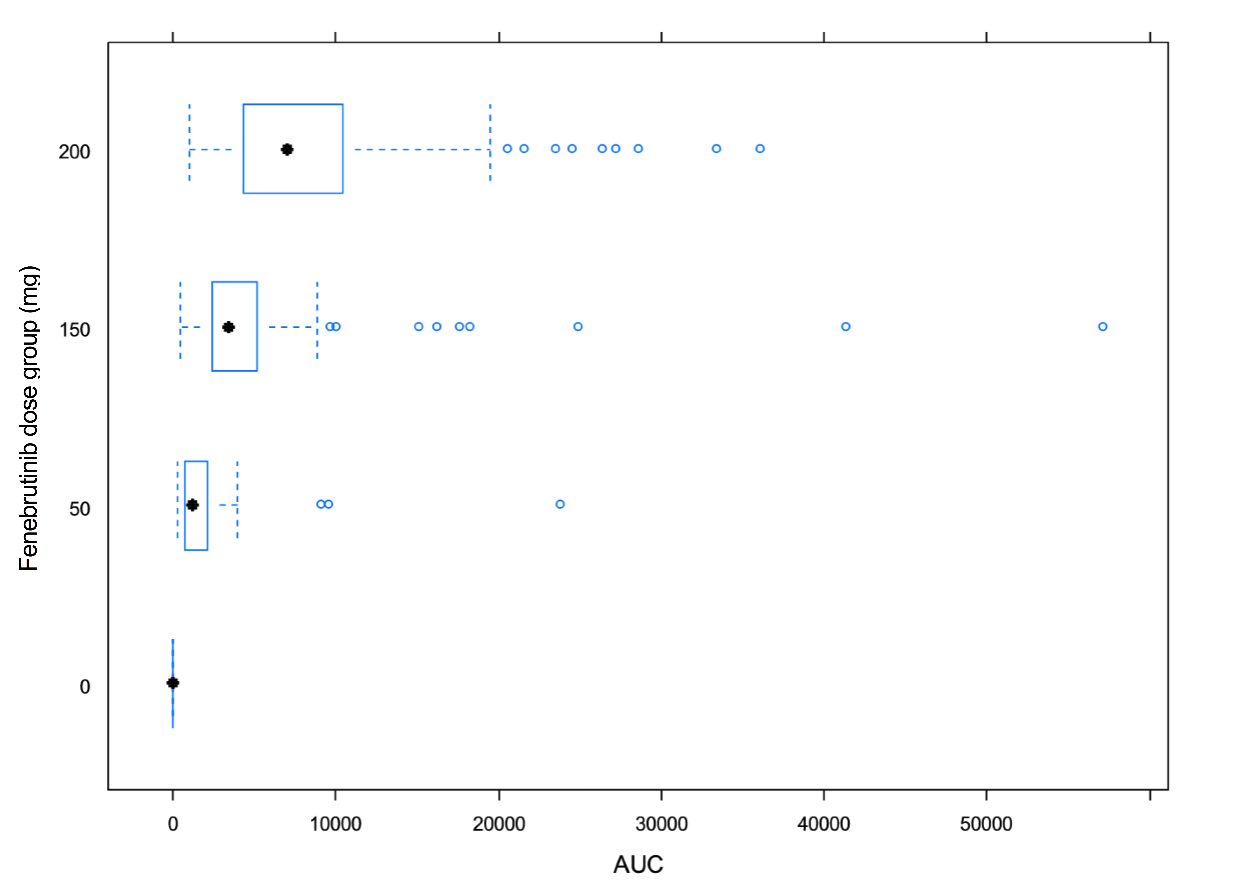


Y-axis, fenebrutinib dose group (mg).

Black Dot: Median; Boxes: Interquartile range;

Whiskers: 1.5 times the interquartile range or the full extent of the data, whichever is less.

AUC, area under plasma-drug concentration curve in 24 hours at steady state (ng/mL∙h).

**Figure S4 ACR20, ACR50, and ACR70 Simulation for Fenebrutinib (GDC-0853), Adalimumab, and Tofacitinib Treatments in TNF-Inhibitor-Inadequate Response Population**

**
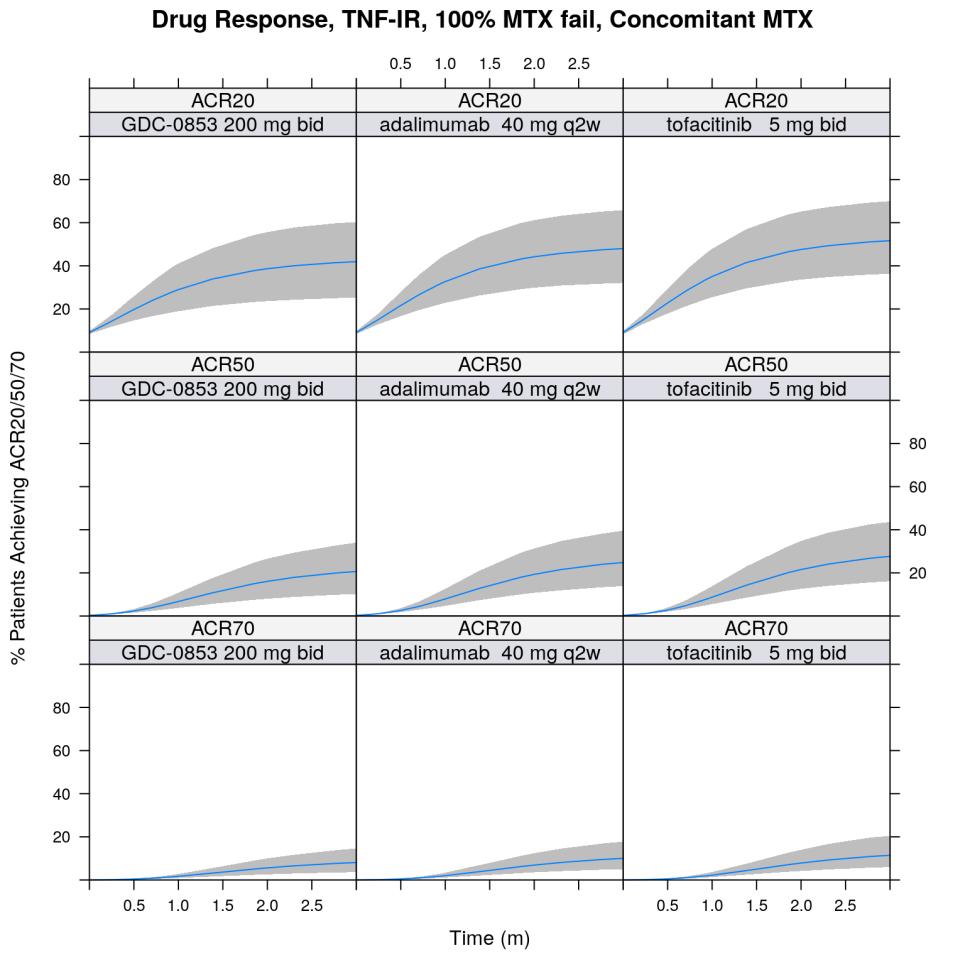
**

Bands: 90% prediction interval; blue line: median prediction

TNF, tumor necrosis factor; IR, inadequate response; MTX, methotrexate;

bid, twice daily; q2w, once every two weeks.

**Model S1: NONMEM Control Stream for Population PK Model**

$SUBROUTINE ADVAN13 TRANS1 TOL=10

$MODEL COMP=(CENTRAL,DEFOBS,DEFDOSE) ;

COMP=(PERIPH) COMP=(PERIPH)

$PK

; =========================== DEFINE OCCASIONS ===================== ;

OC1=0

OC2=0

OC3=0

OC4=0

OC5=0

IF(OCC.EQ.1)OC1=1

IF(OCC.EQ.2)OC2=1

IF(OCC.EQ.3)OC3=1

IF(OCC.EQ.11)OC4=1

IF(OCC.EQ.13)OC5=1

; PTAFD STUDY 350: 144 648 650 653 657 1320 1992

IF(STUDY.EQ.29350.AND.PTAFD.EQ.144) OC1 = 1

IF(STUDY.EQ.29350.AND.PTAFD.EQ.648) OC2 = 1

IF(STUDY.EQ.29350.AND.PTAFD.EQ.650) OC3 = 1

IF(STUDY.EQ.29350.AND.PTAFD.EQ.653) OC3 = 1

IF(STUDY.EQ.29350.AND.PTAFD.EQ.657) OC3 = 1

IF(STUDY.EQ.29350.AND.PTAFD.EQ.1320) OC4 = 1

IF(STUDY.EQ.29350.AND.PTAFD.EQ.1992) OC5 = 1

IOVF1=OC1*ETA(10)+OC2*ETA(11)+OC3*ETA(12)+OC4*ETA(13)+OC5*ETA(14)

; ======= DEFINE COVARIATE RELATIONSHIPS ===========================;

;;; CL-AGE DEFINITION

CLAGE = ((AGE/48)**THETA(22))

;;; CL-PAT DEFINITION

HVPAT = 0 ; patients most common

IF(PAT.LT.1) HVPAT=1 ; for healthy

CLPAT = HVPAT*THETA(23)

;;; CL RELATIONS

CLCOV=EXP(CLAGE)*EXP(CLPAT)

; ============= INITIALIZE TIME AND DOSE FOR TCAM MODELING ========= ;

IDX=1000

IF(STUDY.EQ.29347) IDX=2000

IF(STUDY.EQ.29350) IDX=3000

STRT = IDX+FORM*100+FED*10+PPI

IF(NEWIND.NE.2) COM(3) = 0

IF(NEWIND.NE.2) COM(4) = 0

IF(EVID.EQ.1.AND.AMT.NE.0) COM(3) = AMT

DOS=COM(3)

; CALCULATE DOSE INPUT PARAMETERS

IF(NEWIND.NE.2) COM(1) = TIME

IF(EVID.EQ.1) COM(1) = TIME

; ======= DEFINE INDICATOR VARIABLES FOR PPI USE AND FED STATUS ===== ;

PP=0

IF(PPI.EQ.1) PP=1

FOOD=0

IF(FED.EQ.1.AND.PAT.EQ.0) FOOD=1

TAB=0

IF(FORM.EQ.2) TAB=1

; ======================== MODEL PARAMETERIZATION ================== ;

TVLCL = THETA(1)

TVLV2 = THETA(2)

TVLV3 = THETA(3)

TVLQ3 = THETA(4)

TVLV4 = THETA(5)

TVLQ4 = THETA(6)

TVLNTR1 = THETA(7)

TVLMTT1 = THETA(8)

TVF1 = 1

MU_1 = TVLCL

MU_2 = TVLV2

MU_3 = TVLV3

MU_4 = TVLQ3

MU_5 = TVLV4

MU_6 = TVLQ4

MU_7 = TVLNTR1

MU_8 = TVLMTT1

NTRCOV = EXP(FED*THETA(16))*EXP(TAB*THETA(17))

MTTCOV = EXP(FED*THETA(11))*EXP(PPI*THETA(12))*EXP(PPI*FED*THETA(13))

CL = EXP(MU_1+ETA(1)) * EXP(PPI*THETA(20)) * CLCOV

V2 = EXP(MU_2+ETA(2))

V3 = EXP(MU_3+ETA(3))

Q3 = EXP(MU_4+ETA(4))

V4 = EXP(MU_5+ETA(5))

Q4 = EXP(MU_6+ETA(6))

NTR1 = EXP(MU_7+ETA(7))*NTRCOV

MTT1 = EXP(MU_8+ETA(8))*MTTCOV

F1COV = EXP(PPI*THETA(14))*EXP(PPI*FED*THETA(15))

DOSF1 = 1*EXP(ETA(9)+IOVF1)*F1COV

FRAC = 0

F1 = 0

; ================= RATE CONSTANTS =============== ;

K = CL/V2

K23 = Q3/V2

K32 = Q3/V3

K24 = Q4/V2

K42 = Q4/V4

KTR1 = (NTR1+1)/MTT1

KA = KTR1

; ===== TRANSIT COMPARTMENT ABSORPTION MODELING ===== ;

; Stirling approximation to gamma

X = 0.00001

LOGF1 = 0.5*LOG(NTR1*2*3.1415926+X) + NTR1*LOG(NTR1+X) - NTR1 + LOG(1+1/12/NTR1+1/288/NTR1/NTR1+X)

DOS=COM(3)

; ============== DIFFERENTIAL EQUATIONS ============= ;

$DES

TSTAR = T - COM(1)

IF(TSTAR.LT.0.001) TSTAR = 0.001

IF(DOSE.EQ.0) THEN

INP1 = 0

ELSE

X = 0.00001

INP1 = EXP(LOG(DOS*DOSF1*(1-FRAC)+X) + NTR1*LOG(KTR1*TSTAR+X) + LOG(KTR1+X)- KTR1*TSTAR - LOGF1)

ENDIF

IF(INP1<0) INP1=0.000001

DADT(1)=INP1-K23*A(1)+K32*A(2)-K24*A(1)+K42*A(3)-K*A(1)

DADT(2)=K23*A(1)-K32*A(2)

DADT(3)=K24*A(1)-K42*A(3)

; =================== ERROR MODEL ===================== ;

$ERROR

IF (PAT.EQ.0) THEN

ERRMAX = EXP(THETA(19))

ERRMAX=ERRMAX/(1+ERRMAX)

PROPERR = EXP(THETA(9))*(1-ERRMAX * (1- EXP(-EXP(THETA(18))*TAD)))

ELSE

PROPERR = EXP(THETA(21))

ENDIF

IPRED=A(1)/V2

IRES=DV-IPRED

W=SQRT(PROPERR**2*IPRED**2+THETA(10)**2)

IWRES=IRES/W

Y=IPRED+EPS(1)*W

IF(ICALL.EQ.4.AND.Y.LT.0.001) Y=0.001

**Model S2: NONMEM Control Stream for ACR E-R Model**

$PRED

IF(GEOGUSEUKORLATAM.EQ.1) REGION = 1 ; US

IF(GEOGUSEUKORLATAM.EQ.2) REGION = 2 ; Eastern Europe

IF(GEOGUSEUKORLATAM.EQ.3.OR.GEOGUSEUKORLATAM.EQ.4) REGION = 3 ; Latin america

RF=RFIN

IF(RFIN.EQ.0) RF=124 ; Median RF

IF(REGION.EQ.1) REGCOV = THETA(13) ; Fraction of EMAX

IF(REGION.EQ.2) REGCOV = 1 ; Eastern Europe most common

IF(REGION.EQ.3) REGCOV = THETA(14) ; Fraction of EMAX, Latin America

RFCOV = (RF/124)**THETA(15) ; RF centered on median

;--------------------------------------

IDX=0

IF(DOSE.GT.0) IDX=1

ACR20=0

ACR50=0

ACR70=0

IF(TYPE.EQ.10) ACR20=1

IF(TYPE.EQ.14) ACR50=1

IF(TYPE.EQ.18) ACR70=1

; add markovian element

MARKOV = THETA(4)*PDV

TVBASE=THETA(1)*ACR20+THETA(2)*ACR50+THETA(3)*ACR70

HILL=EXP(THETA(12))

TVLET50=THETA(8)*ACR20+THETA(11)*ACR50+THETA(11)*ACR70

TMAX = THETA(7)*(DAY**HILL)/((DAY**HILL)+EXP(TVLET50)**HILL)

TVLOGIT=TVBASE

DEFF = THETA(9)*REGCOV *RFCOV *AUC/(AUC+EXP(THETA(10)))

;IF(COHORT.EQ.2) DEFF = THETA(11)*AUC/(AUC+EXP(THETA(12)))

LOGIT = TVLOGIT+TMAX+DEFF+MARKOV+ETA(1)

A=EXP(LOGIT)

P=A/(1+A)

IF(P.LT.0.00001) P=0.00001

IF(P.GT.0.99999) P=0.99999

IF (DV.GT.0.5) THEN

Y=P

ELSE

Y=1-P

ENDIF

**Model S3: NONMEM Control Stream for DAS28 E-R Model**

$PRED

EAUC50 = EXP(THETA(5))

TVC = THETA(1)

TVTMAX = THETA(2)+THETA(6)*AUC/(EAUC50+AUC)

LNET50 = THETA(3)

LNED50 = THETA(4)

C = TVC * EXP(ETA(1))

TMAX = TVTMAX * EXP(ETA(2))

ET50 = EXP(LNET50)

ED50 = EXP(LNED50)

THILL = 1

IF(DOSE.GT.0) TMTEFF = (TMAX*(DAY**THILL)) / (DAY**THILL + ED50**THILL)

IF(DOSE.EQ.0) TMTEFF = (TMAX*(DAY**THILL)) / (DAY**THILL + ET50**THILL)

DEFF = 0

IPRED = C + TMTEFF + DEFF

Y = IPRED + EPS(1)

IRES = DV - IPRED

**Data S1:** **R Code for MBMA Model**

nlme.acrxx.4.124 = nlme.run(logit.acrxx ~ base.20*(endpoint=="ACR20") + base.50*(endpoint=="ACR50") +

base.70*(endpoint=="ACR70") +

(acr.f(eff.20,eff.50,eff.70,endpoint) + eta1 + eta2 +

(drug1.name.clean == "abatacept")*(aba.slope*drug1.gendose) +

(drug1.name.clean == "etanercept")*((etan.emax*drug1.gendose)/

(drug1.gendose+exp(etan.ln.ed50))) +

(drug1.name.clean == "ixekizumab")*(ixe.slope*drug1.gendose) +

(drug1.name.clean == "tocilizumab")*(toc.slope*drug1.gendose) +

(drug1.name.clean == "adalimumab")*(ada.emax*drug1.gendose)/

(drug1.gendose + exp(ada.ln.ed50)) +

(drug1.name.clean == "ocrelizumab") * (ocre.const) +

(drug1.name.clean == "tofacitinib") * (tofa.emax*drug1.gendose)/(drug1.gendose + exp(tofa.ln.ed50)) +

(drug1.name.clean == "atacicept") * (ata.const) +

(drug1.name.clean == "GDC-0853") * (gdc.emax*drug1.gendose)/(drug1.gendose + 525) +

(x.tnf.ir.cat == ">80")*tnf.ir.high +

mtx.fail*mtx.fail.slp + con.mtx*con.mtx.eff

) * (1 - exp(-(rate + eta3)*time.clean)),

data = model.ds.2new,

fixed = list(base.20 + base.50 + base.70 + eff.20 + eff.50 + eff.70 + rate + ada.emax +

ada.ln.ed50 + tofa.emax + tofa.ln.ed50 + aba.slope + etan.emax + etan.ln.ed50 +

ixe.slope + toc.slope + ata.const + ocre.const + tnf.ir.high + gdc.emax +

mtx.fail.slp + con.mtx.eff ~ 1),

start = c(base.20=-2.2,base.50 = -4.8, base.70 = -7.7, eff.20=0.5, eff.50=-1, eff.70=-0.4,

rate=1.175, tofa.emax = 2, tofa.ln.ed50 = 4, ada.emax = 1,

ada.ln.ed50 = 1.5, aba.slope = 0.01, etan.emax = 2, etan.ln.ed50 = 2.5,

ixe.slope = 0.01,

toc.slope = 0.01, ata.const = 0.01, ocre.const = 1,

tnf.ir.high = -0.9, gdc.emax = 1, mtx.fail.slp=0.01, con.mtx.eff=0.01),

random = list(reference.id.clean = eta1 ~ 1,

arm.id = eta2 ~ 1,

drug1.name.clean = eta3 ~ 1),

)
